# Self-measurement of blood pressure at home using a cuff device for change in blood pressure levels: systematic review and meta-analysis

**DOI:** 10.1038/s41440-024-01981-4

**Published:** 2024-11-21

**Authors:** Michihiro Satoh, Yukako Tatsumi, Shingo Nakayama, Yukiko Shinohara, Miki Kawazoe, Yoichi Nozato, Ayako Kunimura, Takahisa Murakami, Maya Toyama, Tomoko Muroya, Takahito Yagihashi, Atsushi Sakima, Makiko Abe, Hisatomi Arima, Takayoshi Ohkubo

**Affiliations:** 1 Division of Public Health, Hygiene and Epidemiology, Faculty of Medicine, Tohoku Medical and Pharmaceutical University, Sendai, Japan; 2 Department of Preventive Medicine and Epidemiology, Tohoku Medical Megabank Organization, Tohoku University, Sendai, Japan; 3 Department of Pharmacy, Tohoku Medical and Pharmaceutical University Hospital, Sendai, Japan; 4 Department of Hygiene and Public Health, Teikyo University School of Medicine, Tokyo, Japan; 5 Division of Nephrology and Endocrinology, Faculty of Medicine, Tohoku Medical and Pharmaceutical University, Sendai, Japan; 6 Department of Preventive Medicine & Public Health, Faculty of Medicine, Fukuoka University, Fukuoka, Japan; 7 Department of Geriatric and General Medicine, Osaka University Graduate School of Medicine, Suita, Osaka Japan; 8 Department of Cardiology, Aichi Medical University, Nagakute, Aichi Japan; 9 Izumi Hospital, Sendai, Japan; 10 Health Administration Center, University of the Ryukyus, Nishihara, Okinawa Japan

**Keywords:** Blood pressure monitoring, Ambulatory, Systematic review, Meta-analysis, Telemedicine, Randomized controlled trial

## Abstract

The effect of self-measurement of blood pressure (BP) at home (home BP measurement, HBPM) has been evaluated over the past decade. This meta-analysis included the latest studies to determine whether HBPM reduced BP (PROSPERO ID: CRD42023442225). PubMed, Cochrane Library Database, and IchuShi-Web were searched for randomized controlled trials after the year 2000 which demonstrated the effect of HBPM on BP change compared with usual care (UC). Overall, 65 articles (*n* = 21,053; 63 based on patients with hypertension) were included. The systolic/diastolic BP reduction was significantly greater in the HBPM than in the UC group by 3.27/1.61 mmHg (95% confidence intervals: 2.40–4.15/1.14–2.07) at the end of the intervention, and *I*^2^ values ≥ 46.7% suggested moderate-to-high heterogeneity. The funnel plots exhibited no notable publication bias (Egger’s test *p* ≥ 0.16). HBPM with co-interventions (such as telemonitoring) showed a stronger BP-lowering effect than without co-interventions while the effect of HBPM on BP change remained significant in the absence of co-interventions. HBPM was not associated with systolic BP changes when we combined the four studies that used a wrist cuff device for HBPM. The number of antihypertensive medications increased by 0.17 medications in the HBPM group compared with that in the UC group. There were no significant differences in body mass index changes or risk of severe adverse outcomes between the groups. Our results demonstrated a beneficial effect of HBPM in reducing BP, particularly when used in conjunction with telemonitoring or additional medical support and when employing upper-arm cuff devices.

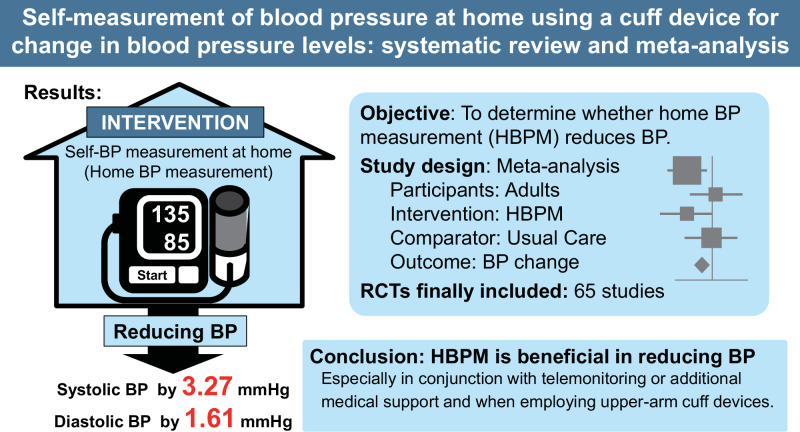

## Introduction

High blood pressure (BP) is a known risk factor for cardiovascular diseases [1–4]. Self-measured BP at home (i.e., home BP) has been shown to predict cardiovascular risks more accurately than conventional BP measured in a medical setting. The Japanese Hypertension Guidelines state that “when there is a discrepancy in diagnosis between office BP and home BP, a home BP-based diagnosis should have priority” [1]. Furthermore, home BP measurement (HBPM) is useful for follow-up of hypertensive patients and recording long-term BP variations (such as seasonal BP). In this context, various home BP monitors have been developed and are currently available. Further development of digital personal health record devices may accelerate the adoption of HBPM as a common healthcare tool.

Previous meta-analyses have revealed that HBPM can lower BP [5–8]. The results of the meta-analyses imply that HBPM alone does not have a large effect [5, 6] and that additional co-interventions (such as telemonitoring) are preferable to achieving an adequate BP-lowering effect [5, 7, 8]. However, several other studies have been published on the effect of self-monitoring of BP since the above-mentioned meta-analyses. Including these recent reports in a meta-analysis allows more accurate estimates to be calculated.

Digital technologies related to human health are developing rapidly and these technologies can sometimes be combined with HBPM, for example, to improve the accuracy of recordings or to make devices wearable. The objective of this meta-analysis was to determine the amplitude of BP reduction after HBPM by including the results of recent reports. Evaluating the magnitude of BP reduction from HBPM can reveal how HBPM contributes to BP management.

## Methods

### Search strategy

This systematic review examined the BP-lowering effects of HBPM compared to usual care (UC). This systematic review was registered in the International Prospective Register of Systematic Reviews known as PROSPERO (ID: CRD42023442225). This systematic review was conducted according to the Preferred Reporting Items for Systematic Reviews and Meta-Analyses (PRISMA) guidelines. This study has used existing, de-identified data from previously published manuscripts or reported on websites, and therefore, was exempted from Institutional Review Board approval. This work is the systematic review for the Task Force “Guideline for BP control using digital technologies” of the Japanese Society of Hypertension.

Studies published in PubMed, Cochrane Library Database, and IchuShi-Web databases were used in this analysis. Studies were included if (1) were reported after the year 2000, (2) were published in English or Japanese, (3) provided the information necessary to calculate estimates and distributions, (4) were randomized controlled trials, or (5) used a cuff device for HBPM. Studies were excluded if (i) were conference abstracts, review articles, case series, qualitative studies, or editorials without any available data, (ii) participants were required to self-measure variables other than BP (e.g., blood glucose) as part of the interventions, (iii) were on pregnant women, (iv) were conducted immediately after a cardiovascular event, and (v) primarily designed to observe the effect of education or other interventions and treated HBPM only as a co-intervention. For studies with overlapping participants, we selected the most recent study with a larger sample size.

The search terms used were combinations of terms related to “self-measured blood pressure” and “randomized controlled trial” as indicated in Supplementary Tables 1–3. The search was performed on August 1, 2023.

### Intervention and comparator

The targeted intervention was self-BP measurement using a cuff device, i.e., HBPM. This included HBPM combined with support from physicians, co-medical professionals, or those using telemonitoring and new technologies (e.g., smartphone applications). The comparator was set as UC without HBPM; however, education or setting the target BP as additional care was allowed if it was considered not to critically affect the present purpose.

### Outcomes

The outcomes were changes in BP, prevention of hypertension, and tapering off BP-lowering medications. However, the latter two outcomes were not often reported in the previous studies. Instead of collecting data on the tapering of BP-lowering medications, we collected data on a change in the number of antihypertensive medications to collect adequate study data. Additional outcomes were adverse events and changes in body mass index (BMI) as a risk factor for cardiovascular diseases. For the outcome of the BP change, we referred to an office or ambulatory BP change because few studies have assessed home BP changes in the UC group.

### Data extraction, selection process, and assessment of bias risk

Data from the included studies were extracted into a standardized form detailing the first author, year of publication, country, study period, population characteristics, study design, intervention, outcomes, sample size, and reported estimates and distributions. When studies reported more than one outcome point, data from the longest intervention period were used for the main analysis. One reviewer extracted the information, and another reviewer confirmed its accuracy.

At each stage, two members of the team independently reviewed the studies. Titles and abstracts were screened during the first screening process. Full texts of relevant articles were sourced in the second screening process, which involved a thorough review of the full texts to ensure the eligibility criteria were met and check for possible repetition of patient data. In cases of disagreement that could not be resolved by consensus, a third reviewer of the review team adjudicated. Two reviewers assessed the potential risk of bias and indirectness of each selected report according to the Minds Manual for Guidelines [9].

### Statistical analysis

The estimates of group differences and 95% confidence intervals (CIs) in BP change at the end of intervention were obtained by fitting random-effects models using restricted maximum likelihood. The 95% CIs were estimated from the standard error (SE) values. When the standard deviation (SD) instead of SE was reported, we computed it as SD/(*n*^0.5^). When the SEs of the differences in BP were not available, we first estimated the SE of the BP difference as [SE_a_^2^ + SE_b_^2^]^0.5^. For example, when we calculated the SE of the BP difference between baseline and follow-up, SE_a_ indicated the SE of the BP at baseline and SE_b_ indicated the SE of the BP at follow-up. If either SE_a_ or SE_b_ were unavailable, the missing data were interpolated based on a regression equation derived from the available data for SE_a_ and SE_b_. Studies without any information on BP distribution were excluded from the analysis. The value obtained at the end of the intervention period was used as BP at follow-up. The present study did not consider changes in BP during observation period after the end of the intervention.

Heterogeneity among the studies was tested using Q-statistics and quantified using *I*^2^ statistics [10]. We considered *I*^2^ < 30% to indicate low heterogeneity between studies, 30%–60% to indicate moderate heterogeneity, and >60% to indicate substantial heterogeneity. Furthermore, the leave-one-out method was used to observe the influence of individual studies on the overall heterogeneity. Funnel plot asymmetry was used to detect publication bias. Egger’s and Begg’s tests were used to examine statistical significance.

Subgroup or meta-regression analyses were used to identify associations between the effect of the intervention on BP change and relevant characteristics including the duration of follow-up, BP measurement methods (office or ambulatory BP), type of device used for the HBPM intervention (wrist or upper arm cuff), co-intervention (present or absent), and change in the number of antihypertensive medications as possible factors of heterogeneity. Co-intervention was defined as the use of telemonitoring, co-medical staff support, or other methods including reminders via telephone or text messages. In meta-regression analyses, when multiple outcome points were found in one study, all data were used after considering the individual studies as random effects. We collected data on office and ambulatory BP as outcomes if the study contained both types of information. When the study had both office and ambulatory BP data, office BP was preferentially considered the main outcome because it was the most common outcome measure.

Analysis was performed using R version 4.4.1 (R Foundation for Statistical Computing, Vienna, Austria) and the R package of “metafor”. Two-sided *p* values of <0.05 were regarded to indicate nominal statistical significance.

## Results

### Study overview

Our search strategy yielded 4378 reports, of which 73 were eligible for a full-text review. Finally, 65 articles were included in the analysis (Supplementary Fig. 1) [11–75]. The characteristics of the included studies are shown in Tables 1 and 2. The study participants were patients with hypertension in 63 of the 65 studies. In one study [14], the previously treated group with SBP/DBP <150/<90 mmHg (*n* = 19 of 40) was not included because of the impracticality of the intervention; in that subgroup, the first attempt was to reduce the number or dosage of antihypertensive medications, resulting in an increase in SBP of ~10 mmHg.

**Table 1:** Summary of basic characteristics

Authors	Year	Country	Hypertension status	Antihypertensive treatment at baseline	Trial name (ID)	Age	Men	*N* of intervention^a^	*N* of control^a^
Vetter et al. [11]	2000	Switzerland	Hypertension	With monotherapy of losartan	SVATCH Study	57.9	49.2%	296	326
Mehos et al. [12]	2000	USA	Hypertension	With antihypertensive medication	–	58.8	30.6%	18	18
Rogers et al. [13]	2001	USA	Hypertension	With or without antihypertensive treatment	–	61.4	49.6%	60	61
Broege et al. [14]	2001	USA	Hypertension	BP > 150/90 mmHg without antihypertensive treatment, age ≥ 65 (the treated group was not considered due to inappropriate protocol)	–	73	32.5%	9	10
Rudd et al. [15]	2004	USA	Hypertension	BP ≥ 150/95 mmHg, with or without antihypertensive treatment	–	59.5	47.0%	74	76
Halme et al. [16]	2005	Finland	Hypertension	With or without antihypertensive treatment	HOMER -	57.2	32.8%	113	119
Zillich et al. [17]	2005	USA	Hypertension	With antihypertensive treatment	HOME -	65.0	39.2%	64	61
Marquez-Contreras et al. [18]	2006	Spain	Hypertension	With or without antihypertensive treatment	EAPACUM–HTA	59.1	51.0%	100	100
Verberk et al. [19]	2007	Netherlands	Hypertension	With or without antihypertensive treatment	HOMERUS	55	55%	214	216
Kauric-Klein and Artinian [20]	2007	USA	Hypertension	With or without antihypertensive treatment, hemodialysis	–	48.7	32%	17	17
Artinian et al. [21]	2007	USA	Hypertension	With or without antihypertensive treatment	–	59.6	36%	194	193
Tobe et al. [22]	2008	Canada	Hypertension	With antihypertensive treatment	IMPPACT	62.9	49%	173	97
Madsen et al. [23]	2008	Denmark	Hypertension	With or without antihypertensive treatment	(NCT00282334)	55.9	50%	113	123
Green et al. [24]	2008	USA	Hypertension	With antihypertensive treatment	e-BP (NCT00158639)	59.1	48%	(1) 259 (2) 261	258
Parati et al. [25]	2009	Italy	Hypertension	With or without antihypertensive treatment	TeleBPCare -	57.5	54%	187	111
da Silva et al. [26]	2009	Brazil	Hypertension	With or without antihypertensive treatment, hemodialysis	([CRG060800146])	51.6	66.2%	34	31
Dejesus et al. [27]	2009	USA	Hypertension	Type 2 diabetes (antihypertensive treatment status is uncertain)	–	>60, 76%	48%	19	17
Bosworth et al. [28]	2009	USA	Hypertension	With or without antihypertensive treatment	TCYB (NCT00123058)	61	34%	158	159
Rinfret et al. [29]	2009	Canada	Hypertension	With or without antihypertensive treatment	(NCT00374829; ISRCTN75436659)	56	54%	111	112
Godwin et al. [30]	2010	Canada	Hypertension	With antihypertensive medications and not at target BP	–	68	49%	285	267
McManus et al. [31]	2010	UK	Hypertension	With antihypertensive treatment	TASMINH2 (ISRCTN17585681)	66	47%	234	246
Varis and Kantola [32]	2010	Finland	Hypertension	Without antihypertensive treatment	–	N/A	39%	104	85
Bosworth et al. [33]	2011	USA	Hypertension	With antihypertensive treatment	HINTS (NCT00237692)	64	16%	149	147
Magid et al. [34]	2011	USA	Hypertension	With antihypertensive treatment	(NCT00520988)	65.9	65%	138	145
Hebert et al. [35]	2012	USA	Hypertension	with antihypertensive treatment self-described black or Hispanic adults	–	61	34%	(1)120 (2)120	(1)129 (2)118
Fuchs et al. [36]	2012	Brazil	Hypertension	With antihypertensive treatment	MONITOR (NCT00921791)	59	40%	62	59
Piette et al. [37]	2012	USA and Mexico	Hypertension	With or without antihypertensive treatment	(NCT01484782)	57.6	33%	89	92
Williams et al. [38]	2012	Australia	Hypertension	With antihypertensive treatment, diabetes, and chronic kidney disease	MESMI (ACTRN12607000044426)	67.0	56%	39	41
Kerry et al. [39]	2013	UK	Hypertension	With or without antihypertensive treatment, Stroke or transient ischemic attack within the 9 months before enrollment and hypertension.	(NCT00514800)	72	57%	187	194
Magid et al. [40]	2013	USA	Hypertension	With or without antihypertensive treatment	(NCT01162759)	60	60%	175	173
McKinstry et al. [41]	2013	UK	Hypertension	With or without antihypertensive treatment	HITS (ISRCTN72614272)	61	59%	218	225
Margolis et al. [42]	2013	USA	Hypertension	With or without antihypertensive treatment	(NCT00781365)	61.1	55%	228	222
Ogedegbe et al. [43]	2014	USA	Hypertension	With antihypertensive treatment	CAATCH (NCT00233220)	56.5	28%	529	510
Stewart et al. [44]	2014	Australia	Hypertension	With antihypertensive treatment (at least one hypertensive medication)	HAPPy -	66.7	51%	207	188
McManus et al. [45]	2014	UK	Hypertension	With high-risk conditions (cardiovascular disease, diabetes, stage 3 chronic kidney disease, or coronary heart disease)	TASMIN-SR -	69.5	60%	276	276
Hosseininasab et al. [46]	2014	Iran	Hypertension	With or without antihypertensive treatment	(NCT01525108)	58.7	38.7%	97	97
Kim et al. [47]	2014	USA	Hypertension	With or without antihypertensive treatment (Korean American aged ≥60 years)	Self-Help Intervention Program on the Control of home BP (NCT00406614)	70.9	30%	184	185
Yi et al. [48]	2015	USA	Hypertension	With antihypertensive treatment	(NCT01123577)	61.3	32%	409	419
Hanley et al. [49]	2015	UK	Hypertension	With antihypertensive treatment (Stroke/TIA survivors with uncontrolled BP)	(ISRCTN61528726)	70.9	60%	40	15
Aekplakorn et al. [50]	2016	Thailand	Hypertension	With antihypertensive treatment	–	59.4	35%	111	113
Kim et al. [51]	2016	USA	Hypertension	With antihypertensive treatment	(NCT01975428)	57.6	32%	52	43
Tzourio et al. [52]	2017	France	Hypertension	With or without antihypertensive treatment, age ≥ 65	(ISRCTN97164929)	79.1	43.0	526	517
Klarskov et al. [53]	2018	Denmark	Hypertension	With or without antihypertensive treatment	BRIDGE	61.8	48.1%	533	515
Martinez et al. [54]	2018	Spain	Hypertension	With antihypertensive treatment	RECAVA	66	64%	64	52
McManus et al. [55]	2018	UK	Hypertension	With antihypertensive treatment	TASMINH4	66.9	53.6%	(1) 391 (2) 389	393
Pan et al. [56]	2018	China	Hypertension	With or without antihypertensive treatment	–	57.2	46.8%	52	55
Skolarus et al. [57]	2018	USA	Hypertension	With or without antihypertensive treatment	Reach Out ED (NCT02664610)	58	21%	48	46
Cuffee et al. [58]	2019	USA	Hypertension	With antihypertensive treatment	([NCT01035554])	58.7	59.2%	106	107
Gu et al. [59]	2020	China	Hypertension	With antihypertensive treatment, diabetes, aged ≥ 60	(UMIN000021613)	71.1	36.7%	45	45
Ojji et al. [60]	2020	Nigeria	Hypertension	With or without antihypertensive treatment	–	44	40%	20	20
Zha et al. [61]	2020	USA	Hypertension	With antihypertensive treatment	(NCT02632838)	52.3	12%	12	13
Ionov et al. [62]	2021	Russia	Hypertension	With antihypertensive treatment	–	47	60%	160	80
McManus et al. [63]	2021	UK	Hypertension	With antihypertensive treatment	HOME BP (ISRCTN13790648)	66	54%	305	317
Zhang et al. [64]	2021	China	Hypertension	With or without antihypertensive treatment	(NCT00670566)	55.1	47%	96	405
Akl et al. [65]	2021	Lebanon	Hypertension	With antihypertensive treatment (uncertain if the study included patients without antihypertensive treatment)	(ISRCTN16450193)	60.2	51%	39	40
Margolis et al. [66]	2022	USA	Hypertension	With or without antihypertensive treatment	Hyperlink 3 (NCT02996565)	60.2	47%	1648	1423
Okoro et al. [67]	2022	Nigeria	No restriction	CKD Stage1-4 with antihypertensive treatment	–	52.2	38%	73	74
Muijsers et al. [68]	2022	Netherlands and Germany	No restriction	Women with a history of preeclampsia and/or HELLP syndrome but without antihypertensive treatment	BP-PRESELF (NCT03228082)	45.4	0%	96	95
Calderón-Anyosa et al. [69]	2023	Peru	Hypertension	With antihypertensive treatment	(NCT03524456)	68.1	32%	20	20
Doogue et al. [70]	2023	UK	Hypertension	Stroke or TIA patients with antihypertensive treatment	TASMIN5S IRL (ISRCTN57946500)	70	80%	10	5
Hoppe et al. [71]	2023	USA	Hypertension	With antihypertensive treatment, age of 18–39 years	the MyHEART randomized clinical trial (NCT03158051)	35	53%	157	159
Leupold et al. [72]	2023	Germany	Hypertension	With antihypertensive treatment	PIA (DRKS00012680)	58	53%	331	305
Martínez-Ibáñez et al. [73]	2023	Spain	Hypertension	With antihypertensive treatment	ADAMPA (NCT03242785)	64.4	47%	156	156
Nejamis et al. [74]	2023	Argentina	Hypertension	With or without antihypertensive treatment	–	58.6	49%	144	148
Ramos-Zavala et al. [75]	2023	México	Hypertension	With antihypertensive treatment	–	58.1	45.5%	94	84

^a^The number of participants at baseline, including the participants that were excluded from the main analysis. *BP blood pressure*

**Table 2:** Summary of methods for intervention and control

Authors	Year	HBPM method: type/method (device)	Outcome BP	Co-intervention 1	Co-intervention 2	Co-intervention 3	Control
Vetter et al. [11]	2000	Wrist/oscillometric (Omron HEM-605)	OBP	None	–	–	Usual care
Mehos et al. [12]	2000	Upper arm/oscillometric (A&D UA-702)	OBP	Education/change in medication	Telephone calls including checking BP on a phone	Pharmacist support	Usual care
Rogers et al. [13]	2001	Upper arm/oscillometric (Welch Allyn 52500)	24 h-ABP	None	Telemonitoring (telecommunication)	–	Usual care
Broege et al. [14]	2001	Upper arm/oscillometric (Omron HEM-702)	OBP, day/night ABP	Setting target BP	Telephone calls including checking BP on a phone	–	Setting target BP
Rudd et al. [15]	2004	Upper arm/oscillometric (A&D UA-751)	OBP	Change in medication	Telephone calls including checking BP on a phone	Nurse support	Usual care
Halme et al. [16]	2005	Upper arm/oscillometric (Omron M4)	OBP/HBP	Setting target BP	–	–	Setting target BP
Zillich et al. [17]	2005	Upper arm/oscillometric (Omron HEM-737A)	OBP	Change in medication	–	Pharmacist support	Usual care
Marquez-Contreras et al. [18]	2006	Upper arm/oscillometric (Omron M4)	OBP	None	–	–	Usual care
Verberk et al. [19]	2007	Upper arm/oscillometric (Omron HEM-705-CP)	OBP, 24 h-/day/night ABP, HBP	Change in medication	–	–	Usual care
Kauric-Klein and Artinian [20]	2007	Upper arm/oscillometric (Omron 1C)	OBP	Other (hemodialysis)	–	–	Education
Artinian et al. [21]	2007	Upper arm/oscillometric (A&D UA-767)	OBP	Education	Telemonitoring	Nurse support	Usual care
Tobe et al. [22]	2008	Upper arm/oscillometric (A&D UA-767P)	OBP	Change in medication	–	–	Usual care
Madsen et al. [23]	2008	Upper arm/semi-automatic oscillometric (Omron 705IT)	Day/night ABP	None	Telemonitoring	–	Usual care
Green et al. [24]	2008	Upper arm/oscillometric (Omron HEM-705-CP)	OBP	Education	(1) Web-site use (2) Web-site use	(1) None (2) Pharmacist support	Usual care
Parati et al. [25]	2009	Upper arm/oscillometric (Tensioday, Tensiomed)	OBP, day ABP	None	Telemonitoring	–	Setting target BP
da Silva et al. [26]	2009	Upper arm/oscillometric (Omron HEM-705-CP)	24 h-ABP/HBP	Change in medication	–	–	Usual care
Dejesus et al. [27]	2009	Upper arm/oscillometric (Life Source UA-767 Plus)	OBP	Education	–	(A class focusing on hypertension in diabetes)	Education (data based on no intervention group was not considered)
Bosworth et al. [28]	2009	Upper arm/oscillometric (Omron HEM 773AC) or wrist/oscillometric (Omron HEM 637)	OBP	None	Mailing BP logs to the center	–	Usual care
Rinfret et al. [29]	2009	Upper arm/digital (Omron HEM-711AC)	24 h-ABP	Education/change in medication	Telemonitoring	–	Usual care
Godwin et al. [30]	2010	Upper arm/oscillometric (A&D UA-767)	24 h-ABP	None	–	–	Usual care
McManus et al. [31]	2010	Upper arm/automated sphygmomanometer (Omron 705IT)	OBP	Change in medication	Telemonitoring	–	Usual care
Varis and Kantola [32]	2010	Upper arm/oscillometric (Omron 1C)	OBP	None	Mailing BP logs to the center	–	Usual care
Bosworth et al. [33]	2011	Upper arm/oscillometric (A&D UA-767PC)	OBP	Change in medication	Telemedicine (Behavioral Management Intervention group was not considered)	Nurse support	Usual care
Magid et al. [34]	2011	Upper arm/oscillometric (A&D UA-767)	OBP	Education	Telemonitoring	Pharmacist support	Usual care
Hebert et al. [35]	2012	Upper arm/oscillometric (Omron HEM-712C)	OBP	(1) None (2) Education	(1) None (2) Telephone calls including checking BP on a phone	(1) None (2) Nurse support	Usual care
Fuchs et al. [36]	2012	Upper arm/oscillometric (Omron HEM-705-CP)	OBP, 24 h-/day/night ABP	Half of them received pharmacist care	–	–	Usual care (but half of them received pharmacist care)
Piette et al. [37]	2012	Unknown device (in-home cuffs for measurement of BP [on Web page of protocol])	OBP	Education	Automated telephone call	–	Usual care
Williams et al. [38]	2012	Upper arm/oscillometric (A&D UA-787)	OBP	Education	–	Nurse support	Usual care
Kerry et al. [39]	2013	Upper arm/oscillometric (Omron M6)	OBP	Education	Telephone calls including checking BP on a phone	Nurse support	Usual care
Magid et al. [40]	2013	Upper arm/oscillometric (Omron HEM-790IT)	OBP	Education/change in medication	Telemonitoring	Pharmacist support	Usual care
McKinstry et al. [41]	2013	Upper arm/oscillometric (Stabil-O-Graph mobil)	Day ABP	None	Telemonitoring	–	Usual care
Margolis et al. [42]	2013	Upper arm/oscillometric (A&D 767PC)	OBP	Education/change in medication	Telemonitoring	Pharmacist support	Education
Ogedegbe et al. [43]	2014	Upper arm/oscillometric (Microlife BP 3AC1-1)	OBP	Education	–	–	Education
Stewart et al. [44]	2014	Upper arm/oscillometric (Omron T9IT)	OBP	Education Change in medication	–	Pharmacist support	Usual care
McManus et al. [45]	2014	Upper arm/oscillometric (Microlife Watch BP Home)	OBP	Change in medication	–	–	Usual care
Hosseininasab et al. [46]	2014	Wrist/oscillometric (Samsung C&T SHB-200w)	OBP	None	–	–	Usual care
Kim et al. [47]	2014	Upper arm/oscillometric (A&D UA-767)	OBP	Education	Telemonitoring	Nurses and nutritionists support	Usual care
Yi et al. [48]	2015	Unknown device but considered to be cuff-type	OBP	Education	Telemonitoring	—	Usual care
Hanley et al. [49]	2015	Upper arm/oscillometric (IEM Stabil-O-graph)	Day ABP	None	Telemonitoring	Nurse support	Usual care
Aekplakorn et al. [50]	2016	Upper arm/oscillometric (Omron HEM-7117)	OBP	None	–	–	Usual care
Kim et al. [51]	2016	Upper arm/oscillometric (Withings BP Monitor)	OBP	Education	Reminder by an App	–	Usual care
Tzourio et al. [52]	2017	Upper arm/oscillometric (Omron M6)	OBP, HBP	None	–	–	Usual care
Klarskov et al. [53]	2018	Upper arm/oscillometric (Microlife BP 3AC1)	OBP, day/night ABP	None	–	–	Usual care
Martinez et al. [54]	2018	Upper arm/oscillometric (Microlife WatchHome)	24 h-/day/night ABP	None	–	–	Usual care
McManus et al. [55]	2018	Upper arm/oscillometric (Omron M10-IT)	OBP	Change in medication	(1) None (2) Telemonitoring	–	Usual care
Pan et al. [56]	2018	Upper arm/oscillometric (“maibobo” RBP3900 and RBP9000)	OBP	Education	Telemonitoring	A technology including an App	Usual care
Skolarus et al. [57]	2018	Upper arm/oscillometric (Omron BP760)	OBP	Education	SMS message to remind BP measurements	–	Usual care
Cuffee et al. [58]	2019	[Upper arm/oscillometric (Carrera Upper Arm BP Monitor)]	24 h-ABP	None	–	–	Usual care Education
Gu et al. [59]	2020	Upper arm/oscillometric (KD598, Andon)	OBP	Other (Pedometer group was not considered)	Telephone calls	–	Education
Ojji et al. [60]	2020	Upper arm/oscillometric (Omron M3 HEM-7131-E)	OBP	None	–	–	Usual care
Zha et al. [61]	2020	Wrist/oscillometric (iHealth BP7 Wireless BP Monitor, iHealth Lab Inc.)	OBP	Education	Telemonitoring	Nurse support	Usual care
Ionov et al. [62]	2021	Unknown but the device had to be listed in the “STRIDE BP” database.	OBP, 24 h-ABP	Change in medication	Telemonitoring	–	Usual care
McManus et al. [63]	2021	Upper arm/oscillometric (Omron M3 monitor)	OBP	Education	Telemonitoring	A technology including an App	Usual care
Zhang et al. [64]	2021	Upper arm/oscillometric (HEM-7051, Omron Healthcare)	OBP	Setting target BP	–	–	Usual care (setting target BP)
Akl et al. [65]	2021	Upper arm/oscillometric (Omron MIT Elite Plus arm monitor)	OBP	Education	–	–	Usual care
Margolis et al. [66]	2022	Upper arm/oscillometric (A&D Medical 767PC)	OBP	Education	Telemonitoring	Pharmacist support	Usual care
Okoro et al. [67]	2022	Upper arm/oscillometric (Chidalex, Joytech Healthcare Co. Ltd)	OBP	Education	Telephone calls including checking BP on a phone	Pharmacist support	Usual care
Muijsers et al. [68]	2022	Upper arm/oscillometric (Withings BP Monitor)	OBP	Education	Telemonitoring	–	Usual care
Calderón-Anyosa et al. [69]	2023	Upper arm/oscillometric (Omron Series 10)	OBP	Change in medication	Telemonitoring	–	Usual care
Doogue et al. [70]	2023	Upper arm/oscillometric (Omron M10-IT)	OBP	Change in medication	Telemonitoring	–	Usual care
Hoppe et al. [71]	2023	Upper arm/oscillometric (Omron, 7 Series)	OBP, 24 h-ABP	Education	Telephone calls including checking BP on a phone	Health coaching	Usual care
Leupold et al. [72]	2023	Upper arm/oscillometric (BOSO® Medicus Family 4)	OBP	Change in medication	Telemonitoring	A technology including an App	Usual care
Martínez-Ibáñez et al. [73]	2023	Upper arm/oscillometric (Omron M3 HEM-7131-E)	OBP	Change in medication	–	–	Usual care
Nejamis et al. [74]	2023	Upper arm/oscillometric (automatic home BP monitor, model unspecified)	OBP	None (peer mentoring arm was excluded)	–	–	Usual care
Ramos-Zavala et al. [75]	2023	Upper arm/oscillometric (Omron HEM-9200T)	OBP	Education	Telemonitoring	–	Usual care

*BP* blood pressure, *OBP* office blood pressure, *ABP* ambulatory blood pressure, *HBP* home blood pressure

The assessment of bias risk and indirectness is presented in Supplementary Table 4. Participants were not blinded in all studies because of the nature of the intervention, which increased performance bias. Twenty-four studies were assessed as having a high bias risk because at least one of the risks was high.

### The effect of the intervention on BP

**Fig. 1:**
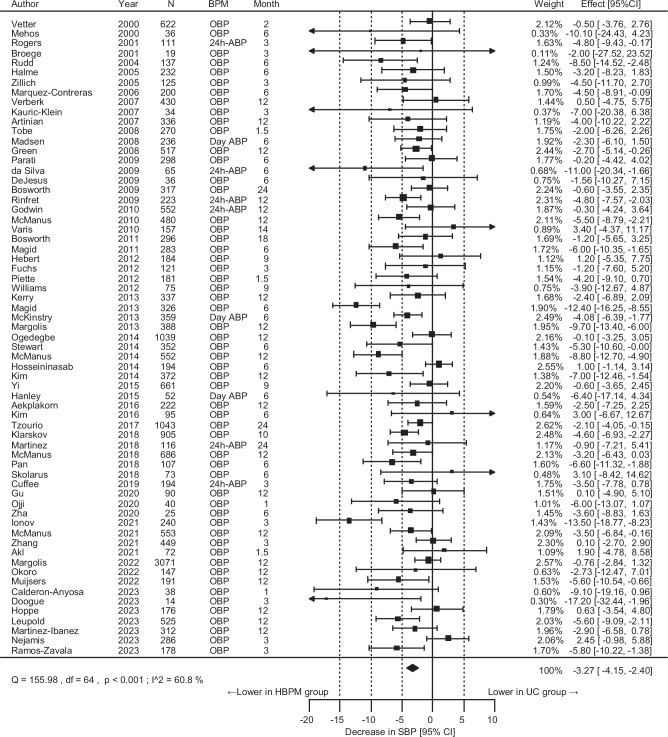
Overall results for the differences in the systolic blood pressure (SBP) change. *N* indicates the final number of participants used for analysis in each study. CI confidence interval, BPM blood pressure measurement method to obtain the outcome value, OBP office blood pressure, ABP ambulatory blood pressure, HBPM home blood pressure measurement, UC usual care

**Fig. 2:**
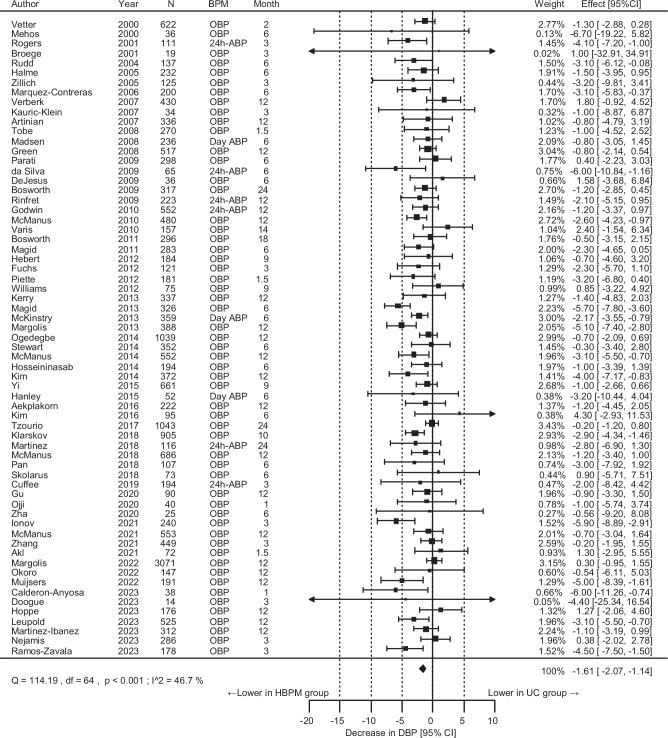
Overall results for the difference in the diastolic blood pressure (DBP) change. *N* indicates the final number of participants used for analysis in each study. CI confidence interval, BPM blood pressure measurement method to obtain the outcome value, OBP office blood pressure, ABP ambulatory blood pressure, HBPM home blood pressure measurement, UC usual care

Of the 65 unique randomized controlled trials, 21,053 participants were included to assess SBP change outcomes. The SBP reduction was significantly greater by 3.27 mmHg at the end of the intervention in the HBPM than in the UC group, although the *I*^2^ value showed high heterogeneity (Fig. 1). Leave-one-out analysis did not identify any specific study to contribute to the high heterogeneity (*I*^2^ value: 53.7–61.6%). The DBP reduction was greater by 1.61 mmHg in the HBPM than in the UC group (Fig. 2, *I*^2^ value: 46.7%). Funnel plots did not exhibit notable publication bias or evidence of publication bias based on Egger’s test (*p* = 0.16 for SBP, *p* = 0.63 for DBP) (Supplementary Fig. 2).

The proportion of participants with controlled BP at the follow-up examination was significantly higher in the HBPM group than in the UC group (proportion rate [95%CI]: 1.24 [1.15–1.34], proportion difference [95%CI]: 0.11% [0.07–0.15], Supplementary Fig. 3) when the sub-analysis was performed in the 30 studies with available data.

### Sensitivity analysis regarding the BP outcome

HBPM intervention was similarly associated with a lower BP change regardless of the type of outcome measure (office or ambulatory BP) (Supplementary Fig. 4 for SBP change and Supplementary Fig. 5 for DBP change). After excluding the 23 studies with a high risk of bias, the result was similar and there was no change in heterogeneity The systolic/diastolic BP change was greater in the HBPM group by 3.47 (95% CI: 2.37–4.56) mmHg (*I*^2^ = 60.9%)/1.72 (95% CI: 1.15–2.28) (*I*^2^ = 43.3%) than the UC group.

To ensure the effect of the follow-up period, a meta-regression analysis was performed based on 97 points of estimates from 65 studies. There was a J-shaped association between follow-up periods and differences in BP changes (Supplementary Fig. 6). The upper limit of the 95% CI became >0 at 20.5 months for the differences in SBP change and 19.5 months for the DBP change, although only three studies indicated the effect of HBPM intervention after 20 months. In an analysis based on the five studies with a follow-up period >12 months, the SBP and DBP changes between groups were lower than the main analysis and became non-significant levels (HBPM minus UC: −1.38 [95% CI: −2.84 to 0.08] mmHg for SBP change and −0.44 [95% CI: −1.23 to 0.34] mmHg for DBP change).

A wrist cuff device was used for the HBPM intervention in four studies (Supplementary Figs. 7 and 8). HBPM intervention was not associated with SBP change when the results from the four studies were combined (−0.06 [95%CI: −1.53 to 1.40] mmHg Supplementary Fig. 7).

**Fig. 3:**
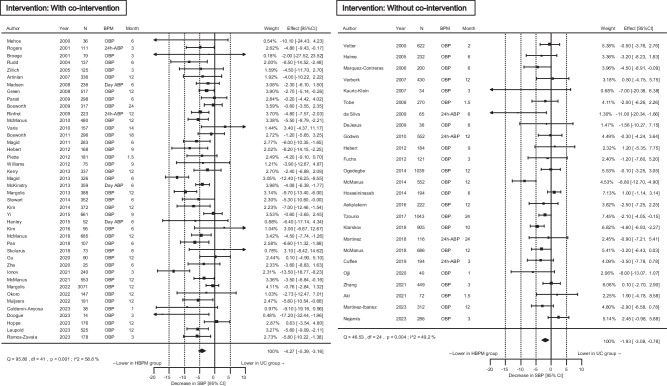
Differences in the systolic blood pressure (SBP) change stratified by co-interventions. Co-intervention indicates support through telemonitoring, co-medical staff, or other methods including reminders via telephone or text message. CI confidence interval, BPM blood pressure measurement method to obtain the outcome value, OBP office blood pressure, ABP ambulatory blood pressure, HBPM home blood pressure measurement, UC usual care

**Fig. 4:**
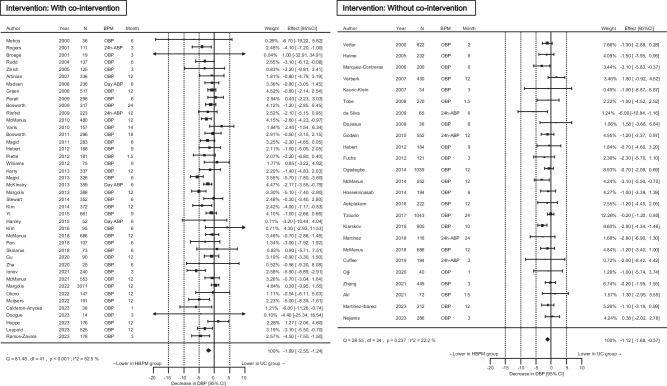
Differences in the diastolic blood pressure (DBP) change stratified by co-interventions. Co-intervention indicates support through telemonitoring, co-medical staff, or other methods including reminders via telephone or text message. CI confidence interval, BPM blood pressure measurement method to obtain the outcome value, OBP office blood pressure, ABP ambulatory blood pressure, HBPM home blood pressure measurement, UC usual care

HBPM analysis with a co-intervention (such as telemonitoring) showed a stronger BP-lowering effect than without co-intervention. The effect of HBPM on BP change remained significant without a co-intervention (Figs. 3 and 4). After further stratification by the type of co-intervention, BP reduction by HBPM was greater in the intervention with telemonitoring or co-medical staff support than in those with other methods including those with only text messages or telephone calls (Supplementary Fig. 9).

### Antihypertensive drug change

Of the 65 studies, 11 reported a change in the number of antihypertensive medications. The number of antihypertensive medications increased by 0.17 medications in the HBPM than in the UC group (Supplementary Fig. 10). The BP-lowering effect of HBPM was more pronounced as the number of antihypertensive medications increased while the meta-regression analysis showed the intercept of the regression slope was −1.72/−1.40 mmHg for SBP/DBP change (*p* = 0.0085/0.012) (Supplementary Fig. 11).

### Other outcomes

We collected information on changes in BMI as a representative index of cardiovascular disease risk factors. No significant difference in BMI change was found when the results of four studies were combined (HBPM minus UC: 0.17 [95%CI: −0.18 to 0.52] kg/m^2^, Supplementary Fig. 12).

Death and cardiovascular disease outcomes were reported as severe adverse events in three studies and five studies, respectively. The analyses based on these studies showed that the risk ratios of HBPM vs UC as a reference for death and cardiovascular diseases were 1.03 (95% CI: 0.63–1.70) and 1.20 (95% CI: 0.68–2.11), respectively, and there were no significant differences between the groups (Supplementary Fig. 13).

## Discussion

The present study demonstrated that HBPM was significantly associated with a larger BP reduction when compared with the UC. A larger BP reduction favoring HBPM was observed when the intervention period was within 20 months, when HBPM was combined with co-interventions such as telemonitoring or co-medical staff support, or when HBPM was performed using an upper-arm cuff device. The meta-analysis had a high heterogeneity but no significant publication bias was observed.

The present meta-analysis revealed that the HBPM can lower SBP/DBP by 3.27/1.61 mmHg more than the UC. HBPM has been recommended for monitoring BP in patients with hypertension because home BP is a stronger predictor of cardiovascular diseases, provides more precise and accurate BP information, and captures longer-term BP or pulse rate variations than office BP [1]. In the previous meta-analyses, HBPM intervention was reported to lower SBP/DBP by 2.63–3.82/1.45–1.68 mmHg compared with UC [5–7]. The values presented in the previous meta-analyses are similar to the present study findings, but the present study estimates the values more accurately by including the latest studies.

A favorable association between HBPM and BP changes appeared to be weakened or enhanced under certain conditions. First, the effect of HBPM may have weakened 20 months after the initiation of the intervention. However, this point should be re-evaluated in the future, as there were only three trials with interventions lasting more than 20 months. Second, the combination of telemonitoring and co-medical staff support can enhance the BP-lowering effect of HBPM, which has been supported by previous meta-analyses [5–7]. The latest individual participant data meta-analysis (IPD) suggested that HBPM alone was not associated with lower BP in the absence of co-interventions [5]. This IPD meta-analysis did not include studies with small sample sizes (*n* < 200). Meanwhile, the present meta-analysis, which evaluated a whole study, suggested that a small but significant favorable BP change could be caused by HBPM, even in the absence of co-interventions. Third, HBPM using a wrist cuff device may not improve the patient’s BP, although the number of reports based on a wrist cuff device was limited. Increasing the sample size may not change this outcome because the effect size of wrist cuff device-based HBPM on systolic BP is almost negligible. Therefore, a thorough review of the protocol may be required to detect the beneficial effects of HBPM using a wrist cuff device. For instance, the use of newly developed wrist cuff devices designed for accurate BP measurement or the implementation of strict patient education may be necessary. An upper arm cuff device has been recommended to obtain accurate BP [1]. The present findings suggest that accurate measurement of BP with an upper arm cuff is critical to obtain the benefits of HBPM at this time.

Our sensitivity analysis suggested that intensifying the antihypertensive treatment might have caused the BP-lowering effect of HBPM. The previous IPD meta-analysis also indicated a correlation between increased number of medication changes and reduced BP, which is similar to the present study results [5]. Home BP-based treatment is superior to office BP-based treatment in achieving the BP target [76]. These findings suggest that HBPM can help identify masked or white-coat uncontrolled hypertension and appropriately adjust for antihypertensive medications. Meanwhile, the meta-regression analysis showed that the intercept of the regression equation between the group difference in the change of antihypertensive medication and the group difference in the BP-lowering effect of HBPM was significantly negative. This intercept reflects the effect of HBPM when the antihypertensive medications were adjusted equally between HBPM and UC groups. Therefore, HBPM per se presumably has a BP-lowering effect even when antihypertensive medications are not changed. Two studies reported that systolic BP tended to decrease more in the HBPM group than in the UC group, despite almost no difference in the change of antihypertensive medication between the groups [39, 58]. Although the effect of HBPM on systolic BP change in each study was not statistically significant [39, 58], the present meta-analysis demonstrated a possible effect of HBPM independent of antihypertensive medication changes by combining the results from these studies. Based on previous studies [77, 78], improved medication adherence or personalized antihypertensive treatment could have contributed to HBPM’s effectiveness. Another potential pathway for HBPM’s effect could be lifestyle improvement through self-feedback of BP levels although our meta-analysis did not reveal significant difference in BMI changes between HBPM and UC groups.

We found no significant differences in the risk of death or cardiovascular diseases as severe outcomes between the HBPM and UC groups. To capture the risks of these outcomes, a long-term follow-up period and more accurate outcome measurements are required. A previous study based on patients treated with antihypertensive medications estimated that reducing systolic BP by 5 mmHg led to a 9% risk reduction of cardiovascular diseases [79]. Based on this evidence [79], a reduction in systolic BP of 3.27 mmHg with HBPM might reduce cardiovascular risks by 6.0%, if our results can be applied to the management of hypertensive patients.

Our study has several limitations. First, heterogeneity was high in most analyses. This may be due to variations in the inclusion criteria, HBPM intervention methods, outcome measures, and follow-up period. If HBPM method is introduced in clinical practice, we should refer to an individual study with a similar intervention method. Second, we could not evaluate the long-term effects of HBPM beyond 1.5 years because of the limited number of trials. Third, owing to the nature of the intervention, the results may be biased based on the Hawthorne effect. However, HBPM intervention is expected to contain the effect by encouraging participants to improve their health behaviors in the first place. It is difficult to distinguish biases such as Hawthorne effect from the effects of HBPM. Fourth, we could not conduct analyses for health behavioral changes to lower BP, adherence to HBPM, and change in the quality of life because of the inconsistent outcomes or missing values among the reports although these were set as additional outcomes in our protocol. One meta-analysis that investigated the effect of HBPM on medication adherence suggested that HBPM intervention may contribute to improved medication adherence [77]. Finally, most studies were based on participants under antihypertensive treatment or populations mixed with treated and untreated individuals. Antihypertensive treatment may have been initiated in participants without prior treatment. Therefore, future studies in non-hypertensive patients who do not require pharmacological therapy may be necessary to assess the impact of HBPM that is not mediated by antihypertensive medications.

## Conclusion

Our results demonstrated a beneficial effect of HBPM in lowering BP, particularly when used in conjunction with telemonitoring or additional medical support, and when measurements are taken with an upper-arm cuff device. Although further research is required to elucidate the long-term effects of HBPM and its impact on severe health outcomes, HBPM is a valuable tool for the treatment of hypertension, potentially enhancing medication adherence and facilitating more personalized and effective management of BP. The development and dissemination of digital technologies for HBPM and support systems can aid in BP management.

## Supplementary information

Supplementary Information

## Data Availability

The authors declare that all supporting data are available in the article and online Supplementary files.
