# Supplementary material for: Self-measurement of blood pressure at home using a cuff device for change in blood pressure levels: systematic review and meta-analysis
Source: Hypertens Res. 2024 Nov 21;48(2):574–91. doi: 10.1038/s41440-024-01981-4 (PMC11794135; doi:10.1038/s41440-024-01981-4)
Supplement: Supplementary file 1 — Supplementary Information [file 41440_2024_1981_MOESM1_ESM.pdf]

Supplementary Information

Supplemental to:

Self-measurement of blood pressure at home using a cuff device for change in blood pressure levels: systematic review and meta-analysis

Supplementary Table 1. Search terms used for PubMed (MEDLINE)

#	Terms	Number
1	(randomized controlled trial[pt] OR controlled clinical trial[pt] OR randomized[tiab] OR placebo[tiab] OR clinical trials as topic[mesh:noexp] OR randomly[tiab] OR trial[ti] NOT (animals[mh] NOT humans [mh]))	1,429,532
2	"Interactive Health Communication Applications"[Title/Abstract]	22
3	"self measurement"[Title/Abstract]	713
4	"self monitoring"[Title/Abstract]	9,288
5	"self-recording"[Title/Abstract]	225
6	"self-performed"[Title/Abstract]	269
7	"home measurement"[Title/Abstract]	133
8	"home intervention"[Title/Abstract]	353
9	"home monitoring"[Title/Abstract]	2,328
10	"remote monitoring"[Title/Abstract]	4,213
11	"telecommunicat*"[Title/Abstract]	5,681
12	"teleconferenc*"[Title/Abstract]	1,574
13	"teleconsult*"[Title/Abstract]	2,424
14	"telemonitor*"[Title/Abstract]	2,628
15	"telehome*"[Title/Abstract]	166
16	"telecare"[Title/Abstract]	1,003
17	"telehealth*"[Title/Abstract]	14,254
18	"telemed*"[Title/Abstract]	25,450
19	"telemetr*"[Title/Abstract]	10,664
20	"Drug Therapy, Computer-Assisted"[Mesh]	1,691
21	"Therapy, Computer-Assisted"[Mesh]	45,700
22	"Internet"[Mesh]	97,614
23	"Computers"[Mesh]	86,495
24	"Home Care Services, Hospital-Based"[Mesh]	1,978
25	"Home Care Services"[Mesh]	50,875
26	"Remote Consultation"[Mesh]	5,815
27	"Telemetry"[Mesh]	15,174
28	"Monitoring, Ambulatory"[Mesh]	31,611
29	"Telecommunications"[Mesh]	124,473
30	"Telemedicine"[Mesh]	44,900
31	#2 OR #3 OR #4 OR #5 OR #6 OR #7 OR #8 OR #9 OR #10 OR #11 OR #12 OR #13 OR #14 OR #15 OR #16 OR #17 OR #18 OR #19 OR #20 OR #21 OR #22 OR #23 OR #24 OR #25 OR #26 OR #27 OR #28 OR #29 OR #30	442,783
32	"Blood Pressure"[Mesh]	309,257
33	"Hypertension"[Mesh]	317,477
34	"Blood Pressure"[Title/Abstract]	345,998
35	"hypertens*"[Title/Abstract]	514,619
36	"Blood Pressure Monitoring, Ambulatory"[Mesh]	11,726
37	"Blood Pressure Monitors"[Mesh]	2,407
38	#32 OR #33 OR #34 OR #35 OR #36 OR #37	895,072
39	("2000/01/01"[Date - Publication] : "2023/07/31"[Date - Publication])	22,450,029
40	#1 and #31 and #38 and #39	3,495

Supplementary Table 2. Search terms used for Cochran database

#	Terms	Number
1	(randomized controlled trial[pt] OR controlled clinical trial[pt] OR randomized[tiab] OR placebo[tiab] OR clinical trials as topic[mesh:noexp] OR randomly[tiab] OR trial[ti] NOT (animals[mh] NOT humans [mh]))	1,304,649
2	"Interactive Health Communication Applications"[Title/Abstract]	5
3	"self measurement"[Title/Abstract]	169
4	"self monitoring"[Title/Abstract]	4,131
5	"self-recording"[Title/Abstract]	51
6	"self-performed"[Title/Abstract]	95
7	"home measurement"[Title/Abstract]	33
8	"home intervention"[Title/Abstract]	329
9	"home monitoring"[Title/Abstract]	577
10	"remote monitoring"[Title/Abstract]	943
11	"telecommunicat*"[Title/Abstract]	0
12	"teleconferenc*"[Title/Abstract]	0
13	"teleconsult*"[Title/Abstract]	8
14	"telemonitor*"[Title/Abstract]	17
15	"telehome*"[Title/Abstract]	15
16	"telecare"[Title/Abstract]	223
17	"telehealth*"[Title/Abstract]	2,340
18	"telemed*"[Title/Abstract]	15
19	"telemetr*"[Title/Abstract]	0
20	"Drug Therapy, Computer-Assisted"[Mesh]	167
21	"Therapy, Computer-Assisted"[Mesh]	2,776
22	"Internet"[Mesh]	6,200
23	"Computers"[Mesh]	2,647
24	"Home Care Services, Hospital-Based"[Mesh]	268
25	"Home Care Services"[Mesh]	2,836
26	"Remote Consultation"[Mesh]	441
27	"Telemetry"[Mesh]	377
28	"Monitoring, Ambulatory"[Mesh]	3,680
29	"Telecommunications"[Mesh]	10,844
30	"Telemedicine"[Mesh]	4,268
31	#2 OR #3 OR #4 OR #5 OR #6 OR #7 OR #8 OR #9 OR #10 OR #11 OR #12 OR #13 OR #14 OR #15 OR #16 OR #17 OR #18 OR #19 OR #20 OR #21 OR #22 OR #23 OR #24 OR #25 OR #26 OR #27 OR #28 OR #29 OR #30	32,395
32	"Blood Pressure"[Mesh]	33,918
33	"Hypertension"[Mesh]	26,563
34	"Blood Pressure"[Title/Abstract]	92,175
35	"hypertens*"[Title/Abstract]	27
36	"Blood Pressure Monitoring, Ambulatory"[Mesh]	1,788
37	"Blood Pressure Monitors"[Mesh]	167
38	#32 OR #33 OR #34 OR #35 OR #36 OR #37	112,371
39	("2000/01/01"[Date - Publication] : "3000"[Date - Publication])	3,277
40	#1 and #31 and #38 and #39	2,953

Supplementary Table 3. Search terms used for Ichu-shi (in Japanese)

#	検索式	文献数
1	(ランダム化比較試験/TH or ランダム化比較試験/TA or RCT/TA or 準ランダム化比較試験/TH)	77,351
2	自己測定/TA	1,866
3	自己監視/TA	28
4	自己記録/TA	106
5	自己実施/TA	5
6	自宅測定/TA	7
7	自宅介入/TA	1
8	自宅監視/TA	0
9	リモート/TA or 遠隔/AL	60,445
10	"テレ*"/TA	0
11	"コンピュータ支援薬物療法"/TH	82
12	"コンピュータ支援治療法"/TH	10,713
13	"インターネット"/TH	26,850
14	"コンピュータ"/TH	20,149
15	"病院基盤在宅介護支援サービス"/TH	837
16	"在宅介護支援サービス"/TH	56,336
17	"遠隔診療"/TH	7,068
18	"テレメトリー"/TH	2,760
19	"自由行動下血圧測定"/TH	2,608
20	"遠隔通信"/TH	33,958
21	"遠隔医療"/TH	12,820
22	"血圧"/TH	46,336
23	"高血圧"/TH	130,278
24	"血圧"/TA	201,588
25	"高血圧"/TA	134,263
26	"家庭血圧測定"/TH	330
27	"血圧計"/TH	2,075
28	(PDAT=2000/01/01:2023/07/31)	15,837,978
29	#2 or #3 or #4 or #5 or #6 or #7 or #8 or #9 or #10 or #11 or #12 or #13 or #14 or #15 or #16 or #17 or #18 or #19 or #20 or #21	185,119
30	#22 or #23 or #24 or #25 or #26 or #27	251,805
31	#1 and #28 and #29 and #30	189

Supplementary Table 4. Risk of Bias and Indirectness

		Risk of bias										Indirectness				
		Selection bias		Performance bias	Detection bias	Attrition bias		Other bias								
First author	Year	Random sequence generation	Allocation concealment	Blinding of participants and personnel	Blinding of outcome assessment	Intention-to-treat analysis	Incomplete outcome data	Selective outcome reporting	Early termination	Others	Summary	Population,	intervention,	comparator	outcomes	Summary
Vetter	2000	-1	-1	-1	0	-2	-2	-1	-1	-1	-2	-1	-1	-1	0	-1
Mehos	2000	-1	-1	-1	-1	-1	0	-1	-1	-1	-1	0	-1	0	0	-1
Rogers	2001	0	0	-1	0	-1	0	-1	0	-1	-1	0	-1	0	-1	-1
Broege	2001	-1	-1	-1	0	-1	-1	-1	-1	0	-1	-1	-2	-2	0	-2
Rudd	2004	0	-1	-1	0	-1	-1	-1	-1	0	-1	0	-1	0	0	-1
Halme	2005	-1	-1	-1	0	-1	0	-1	0	0	-1	0	0	-1	0	-1
Zillich	2005	-2	-1	-1	-1	-1	0	-1	0	0	-2	-1	-1	0	0	-1
Marquez-Contreras	2006	0	0	-1	-1	-1	0	-1	0	0	-1	0	0	0	0	0
Verberk	2007	0	0	-1	0	0	0	0	0	0	-1	0	-1	-1	0	-1
Kauric-Klein	2007	0	0	-1	0	0	0	-1	0	0	-1	0	-1	-1	0	-2
Artinian	2007	0	0	-1	0	0	-1	-1	-1	0	-1	-1	-1	-1	-1	-1
Tobe	2008	-2	-1	-1	-1	-2	-2	-1	0	0	-2	0	-1	-1	0	-1
Madsen	2008	0	0	-1	-1	0	0	-1	0	0	-1	0	-1	0	0	-1
Green	2008	0	-1	-1	0	0	0	-1	0	0	-1	0	0	0	0	0
Parati	2009	-1	-1	-1	-1	-1	-2	-1	0	0	-2	0	-1	0	0	-1
da Silva	2009	-1	-1	-1	-1	-2	-2	-1	0	0	-2	-2	-1	-1	0	-2
Dejesus	2009	-1	-1	-1	-1	0	-1	-1	-2	0	-2	-1	0	0	0	-1
Bosworth	2009	-1	-1	-1	0	0	0	-1	0	0	-1	0	0	0	0	0
Rinfret	2009	-1	-1	-1	-1	0	-1	0	0	0	-1	0	-1	0	0	-1
Godwin	2010	-2	0	-1	-1	-1	-1	-1	-1	0	-2	0	-1	0	0	-1
McManus	2010	0	0	-1	-1	-1	-1	0	-1	0	-1	0	-1	0	0	-1
Varis	2010	0	0	-1	-1	-1	-1	-1	-1	0	-1	-2	0	-1	0	-2
Bosworth	2011	-1	-1	-1	0	-1	0	0	0	0	-1	0	-1	0	0	-1
Magid	2011	0	-1	-1	0	-1	0	0	0	0	-1	0	-1	0	0	-1
Hebert	2012	0	0	-1	0	-1	0	-1	0	-1	-1	-1	0	0	0	-1
Fuchs	2012	0	0	-1	-1	-1	0	0	0	0	-1	0	-1	-1	0	-1
Piette	2012	0	0	-1	-1	-1	0	-1	0	0	-1	0	-1	0	0	-1
Williams	2012	0	0	-1	-1	-1	0	-1	0	0	-1	-2	-1	0	0	-2
Kerry	2013	0	0	-1	0	-1	0	-1	0	-1	-1	-2	0	-2	0	-2
Magid	2013	0	0	-1	0	-1	0	-1	0	0	-1	0	0	0	0	0
McKinstry	2013	0	0	-1	0	-1	0	-1	0	0	-1	0	-1	0	0	-1
Margolis	2013	-2	-1	-1	-1	-1	0	0	0	0	-2	0	-1	-1	0	-1
Ogedegbe	2014	-2	-1	-1	-1	-1	0	-1	0	0	-2	0	-1	0	0	-1
Stewart	2014	-2	-1	-1	-1	-1	0	0	0	0	-2	0	-1	0	0	-1
McManus	2014	0	-1	-1	-1	-1	0	-1	0	0	-1	-2	-1	-2	0	-2
Hosseininasab	2014	-2	0	-1	0	-1	0	0	0	0	-2	0	0	0	0	0
Kim	2014	-2	-1	-1	-1	-1	0	-1	0	0	-2	-1	-1	0	0	-1
Yi	2015	0	0	-1	-1	-1	-1	0	0	0	-1	0	-1	0	-1	-1
Hanley	2015	-1	-1	-1	-1	-2	0	0	0	0	-2	-2	-1	0	0	-2
Aekplakorn	2016	-2	0	-1	0	-1	0	-1	0	0	-2	0	0	-1	0	-1
Kim	2016	-1	-1	-1	-1	-1	-1	0	-1	0	-1	0	-2	0	0	-2
Tzourio	2017	-2	-2	-1	0	-1	0	0	-1	-1	-2	-1	0	0	0	-1
Klarskov	2018	0	0	-1	0	0	-1	-1	0	0	-1	0	0	0	0	0
Martinez	2018	0	0	-1	0	0	-1	-1	0	0	-1	-2	0	0	0	-2
McManus	2018	0	0	-1	0	0	0	0	0	0	-1	0	0	0	0	0
Pan	2018	0	0	-1	0	0	0	-1	0	0	-1	0	0	0	0	-1
Skolarus	2018	0	0	-1	0	-2	-1	0	0	0	-2	0	0	0	0	0
Cuffee	2019	0	0	-1	0	-1	0	0	0	0	-1	0	-1	-1	0	-1
Gu	2020	0	-1	-1	0	0	0	0	0	0	-1	-2	0	0	0	-2
Ojji	2020	-1	-1	-1	0	0	0	-1	-2	0	-2	0	-1	-1	0	-1
Zha	2020	-1	-1	-1	0	-2	0	0	-2	0	-2	0	0	0	0	0
Ionov	2021	0	0	-1	0	-1	-1	-1	0	0	-1	0	-1	0	0	-1
McManus	2021	0	-1	-1	-1	-1	-1	0	0	0	-1	0	-1	0	0	-1
Zhang	2021	-1	-1	-1	0	0	0	0	0	0	-1	0	-1	-1	0	-1
Akl	2021	0	0	-1	-1	-2	0	0	-2	-1	-2	0	0	0	-2	-2
Margolis	2022	-2	-2	-1	0	0	0	-1	0	0	-2	0	-2	-1	0	-2
Okoro	2022	0	0	-1	0	0	0	-1	0	-1	-1	-2	-2	0	-2	-2
Muijsers	2022	0	0	-1	-1	-1	-1	0	0	0	-1	-2	0	0	0	-2
Calderón-Anyosa	2023	-1	-1	-1	0	-1	0	0	0	0	-1	0	-1	0	0	-1
Doogue	2023	0	0	-1	0	-1	0	-1	-2	0	-2	-2	0	0	0	-2
Hoppe	2023	0	0	-1	0	0	-1	0	0	-1	-1	-1	-1	-1	0	-1
Leupold	2023	-2	0	-1	0	-1	-2	0	0	0	-2	0	-2	0	0	-2
Martínez-Ibáñez	2023	0	0	-1	0	0	-1	-1	-1	0	-1	0	-1	-1	0	-1
Nejamis	2023	0	0	-1	0	0	0	-1	0	0	-1	0	0	0	0	0
Ramos-Zavala	2023	0	-2	-1	0	-1	-1	-1	0	0	-2	0	-1	0	0	-1

The Cochrane Risk of Bias Tool was used to evaluate the studies.

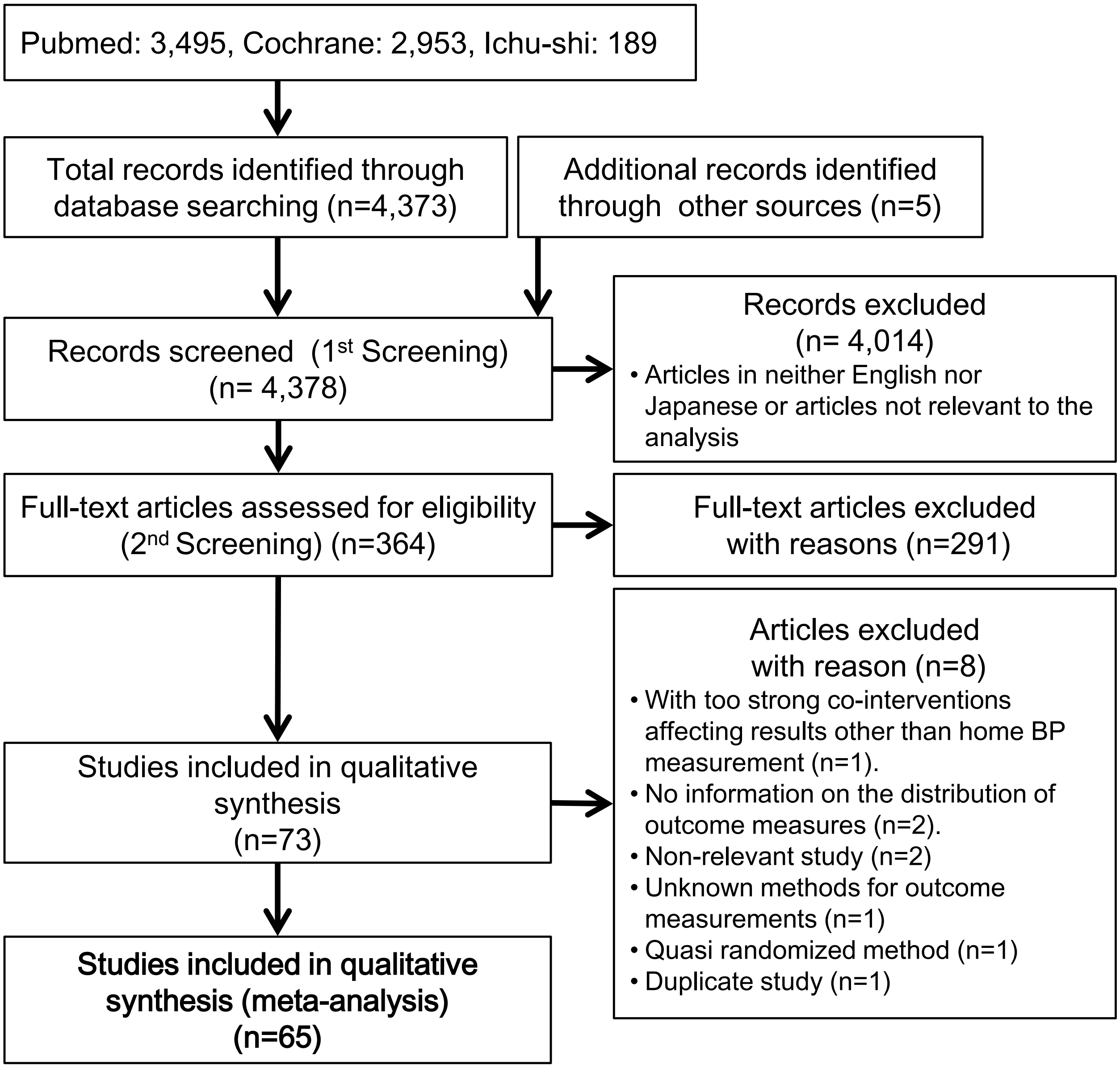

Supplementary Figure 1. Selection of studies for inclusion in the review.

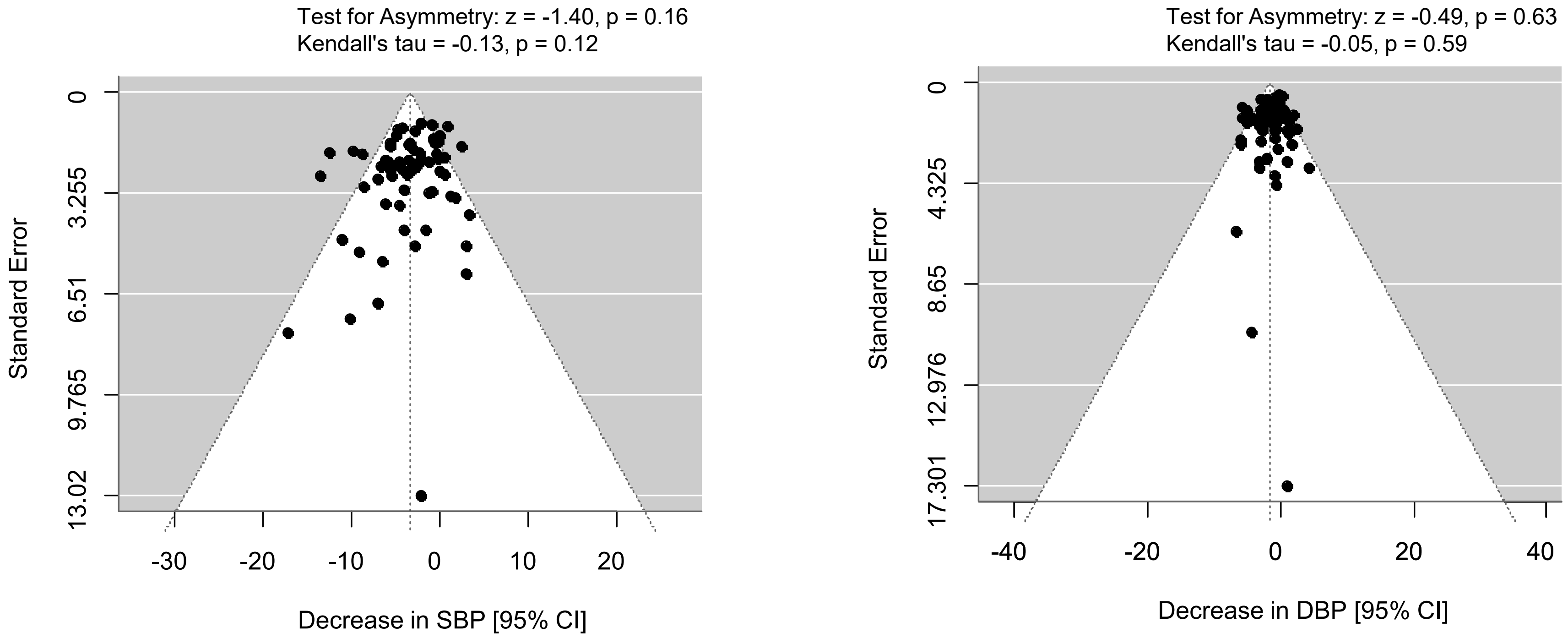

Supplementary Figure 2. Funnel Plot of the differences in systolic (SBP) and diastolic (DBP) blood pressure changes

"Test for Asymmetry" and "Kendall's tau" indicate the results of Egger's test and Begg's test, respectively.

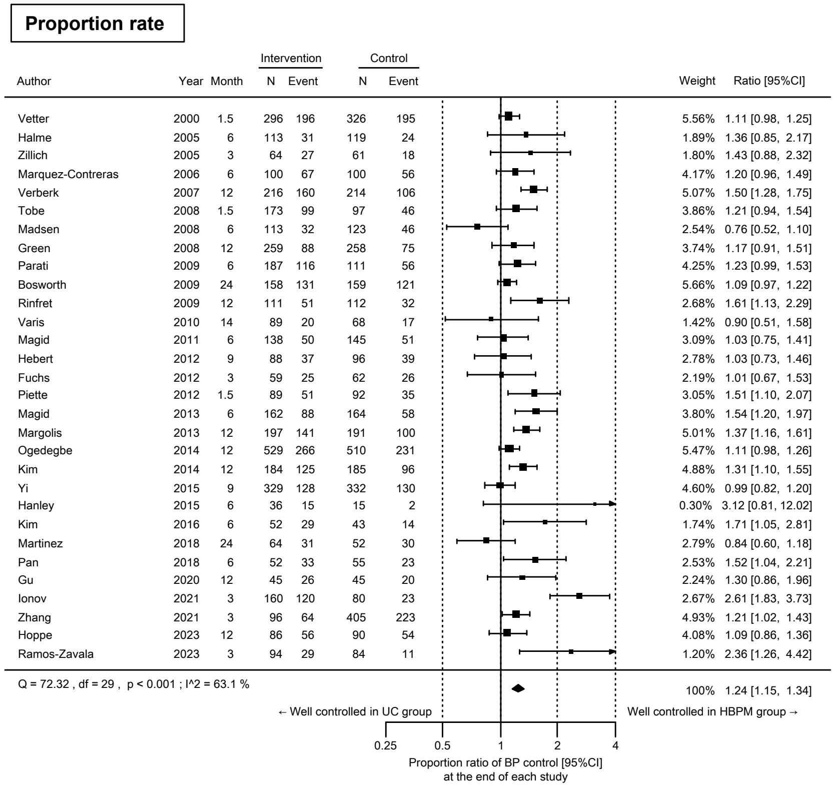

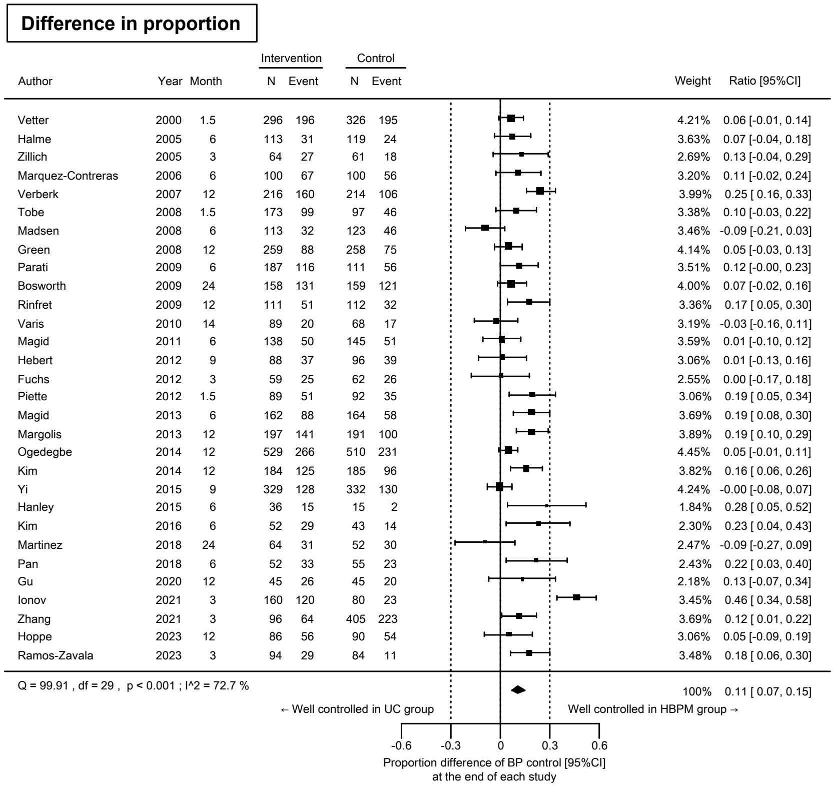

Supplementary Figure 3. Difference in BP control at follow-up between groups

CI, confidence interval; BP, blood pressure; HBPM, home blood pressure measurement; UC, usual care

Outcome measure: Office BP (OBP)

Author	Year	N	BPM	Month	Weight	Effect [95%CI]
Vetter	2000	622	OBP	2	2.43%	-0.50 [-3.76, 2.76]
Mehos	2000	36	OBP	6	0.42%	-10.10 [-24.43, 4.23]
Broege	2001	19	OBP	3	0.14%	-2.00 [-27.52, 23.52]
Rudd	2004	137	OBP	6	1.49%	-8.50 [-14.52, -2.48]
Halme	2005	232	OBP	6	1.78%	-3.20 [-8.23, 1.83]
Zillich	2005	125	OBP	3	1.21%	-4.50 [-11.70, 2.70]
Marquez-Contreras	2006	200	OBP	6	2.00%	-4.50 [-8.91, -0.09]
Verberk	2007	430	OBP	12	1.71%	0.50 [-4.75, 5.75]
Kauric-Klein	2007	34	OBP	3	0.47%	-7.00 [-20.38, 6.38]
Artinian	2007	336	OBP	12	1.44%	-4.00 [-10.22, 2.22]
Tobe	2008	270	OBP	1.5	2.05%	-2.00 [-6.26, 2.26]
Green	2008	517	OBP	12	2.75%	-2.70 [-5.14, -0.26]
Parati	2009	298	OBP	6	2.07%	-0.20 [-4.42, 4.02]
DeJesus	2009	36	OBP	6	0.93%	-1.56 [-10.27, 7.15]
Bosworth	2009	317	OBP	24	2.55%	-0.60 [-3.55, 2.35]
McManus	2010	480	OBP	12	2.42%	-5.50 [-8.79, -2.21]
Varis	2010	157	OBP	14	1.09%	3.40 [-4.37, 11.17]
Bosworth	2011	296	OBP	18	1.98%	-1.20 [-5.65, 3.25]
Magid	2011	283	OBP	6	2.02%	-6.00 [-10.35, -1.65]
Hebert	2012	184	OBP	9	1.35%	1.20 [-5.35, 7.75]
Fuchs	2012	121	OBP	3	1.39%	-1.20 [-7.60, 5.20]
Piette	2012	181	OBP	1.5	1.83%	-4.20 [-9.10, 0.70]
Williams	2012	75	OBP	9	0.92%	-3.90 [-12.67, 4.87]
Kerry	2013	337	OBP	12	1.97%	-2.40 [-6.89, 2.09]
Magid	2013	326	OBP	6	2.20%	-12.40 [-16.25, -8.55]
Margolis	2013	388	OBP	12	2.26%	-9.70 [-13.40, -6.00]
Ogedegbe	2014	1039	OBP	12	2.47%	-0.10 [-3.25, 3.05]
Stewart	2014	352	OBP	6	1.70%	-5.30 [-10.60, -0.00]
McManus	2014	552	OBP	12	2.18%	-8.80 [-12.70, -4.90]
Hosseininasab	2014	194	OBP	6	2.85%	1.00 [-1.14, 3.14]
Kim	2014	372	OBP	12	1.65%	-7.00 [-12.46, -1.54]
Yi	2015	661	OBP	9	2.51%	-0.60 [-3.65, 2.45]
Aekplakorn	2016	222	OBP	12	1.88%	-2.50 [-7.25, 2.25]
Kim	2016	95	OBP	6	0.80%	3.00 [-6.67, 12.67]
Tzourio	2017	1043	OBP	24	2.92%	-2.10 [-4.05, -0.15]
Klarskov	2018	905	OBP	10	2.78%	-4.60 [-6.93, -2.27]
McManus	2018	686	OBP	12	2.44%	-3.20 [-6.43, 0.03]
Pan	2018	107	OBP	6	1.89%	-6.60 [-11.32, -1.88]
Skolarus	2018	73	OBP	6	0.61%	3.10 [-8.42, 14.62]
Gu	2020	90	OBP	12	1.79%	0.10 [-4.90, 5.10]
Ojji	2020	40	OBP	1	1.23%	-6.00 [-13.07, 1.07]
Zha	2020	25	OBP	6	1.72%	-3.60 [-8.83, 1.63]
Ionov	2021	240	OBP	3	1.71%	-13.50 [-18.77, -8.23]
McManus	2021	553	OBP	12	2.40%	-3.50 [-6.84, -0.16]
Zhang	2021	449	OBP	3	2.61%	0.10 [-2.70, 2.90]
Akl	2021	72	OBP	1.5	1.32%	1.90 [-4.78, 8.58]
Margolis	2022	3071	OBP	12	2.88%	-0.76 [-2.84, 1.32]
Okoro	2022	147	OBP	12	0.79%	-2.73 [-12.47, 7.01]
Muijsers	2022	191	OBP	12	1.81%	-5.60 [-10.54, -0.66]
Calderon-Anyosa	2023	38	OBP	1	0.75%	-9.10 [-19.16, 0.96]
Doogue	2023	14	OBP	3	0.38%	-17.20 [-32.44, -1.96]
Hoppe	2023	176	OBP	12	2.08%	0.63 [-3.54, 4.80]
Leupold	2023	525	OBP	12	2.34%	-5.60 [-9.09, -2.11]
Martinez-Ibanez	2023	312	OBP	12	2.27%	-2.90 [-6.58, 0.78]
Nejamis	2023	286	OBP	3	2.36%	2.45 [-0.98, 5.88]
Ramos-Zavala	2023	178	OBP	3	1.99%	-5.80 [-10.22, -1.38]
Q = 146.73 , df = 55 , p < 0.001 ; I^2 = 64.5 %					100%	-3.23 [-4.23, -2.24]

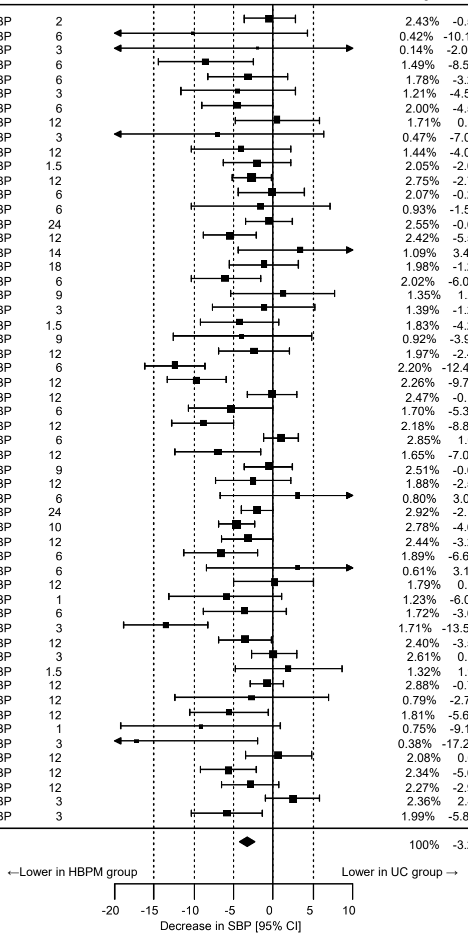

Outcome measure: Ambulatory BP (ABP)

Author	Year	N	BPM	Month	Weight	Effect [95%CI]
Rogers	2001	111	24h-ABP	1	5.94%	-4.80 [-9.43, -0.17]
Broege	2001	19	Day ABP	3	0.69%	-6.00 [-26.06, 14.06]
Verberk	2007	430	24h-ABP	12	7.54%	1.80 [-1.48, 5.08]
Madsen	2008	236	Day ABP	6	6.90%	-2.30 [-6.10, 1.50]
Parati	2009	298	Day ABP	6	7.20%	-1.60 [-5.15, 1.95]
da Silva	2009	65	24h-ABP	6	2.58%	-11.00 [-20.34, -1.66]
Rinfret	2009	223	24h-ABP	12	8.17%	-4.80 [-7.57, -2.03]
Godwin	2010	552	24h-ABP	12	6.74%	-0.30 [-4.24, 3.64]
Fuchs	2012	121	24h-ABP	3	6.08%	-5.40 [-9.90, -0.90]
McKinstry	2013	359	Day ABP	6	8.73%	-4.08 [-6.39, -1.77]
Hanley	2015	52	Day ABP	6	2.07%	-6.40 [-17.14, 4.34]
Klarskov	2018	905	Day ABP	10	9.37%	-0.40 [-2.13, 1.33]
Martinez	2018	116	24h-ABP	24	4.35%	-0.90 [-7.21, 5.41]
Cuffee	2019	194	24h-ABP	3	6.56%	-3.50 [-7.59, 0.59]
Ionov	2021	240	24h-ABP	3	10.02%	-8.80 [-9.77, -7.83]
Hoppe	2023	173	24h-ABP	12	7.05%	-0.51 [-4.19, 3.17]
Q = 123.92 , df = 15 , p < 0.001 ; I^2 = 76.9 %					100%	-3.20 [-4.93, -1.47]

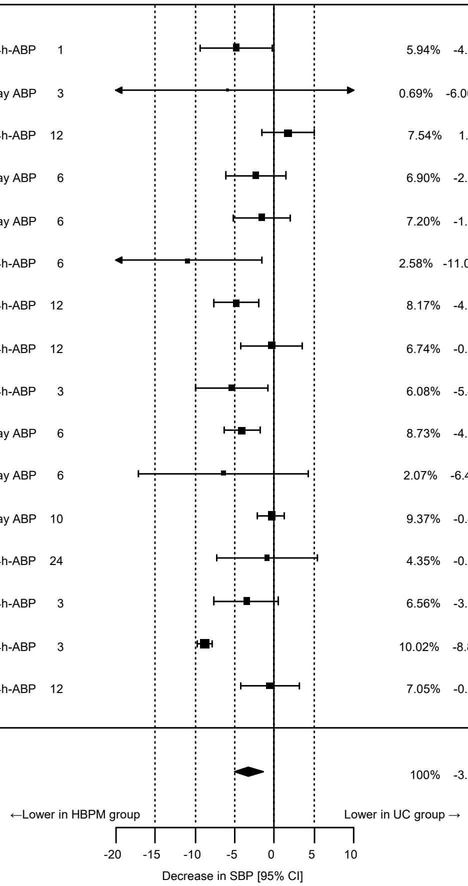

Supplementary Figure 4. The results for SBP change stratified by the type of outcome BP measurements

CI, confidence interval; BPM, blood pressure measurement method to obtain the outcome value; OBP, office blood pressure; ABP, ambulatory blood pressure; HBPM, home blood pressure measurement; UC, usual care

Outcome measure: Office BP (OBP)

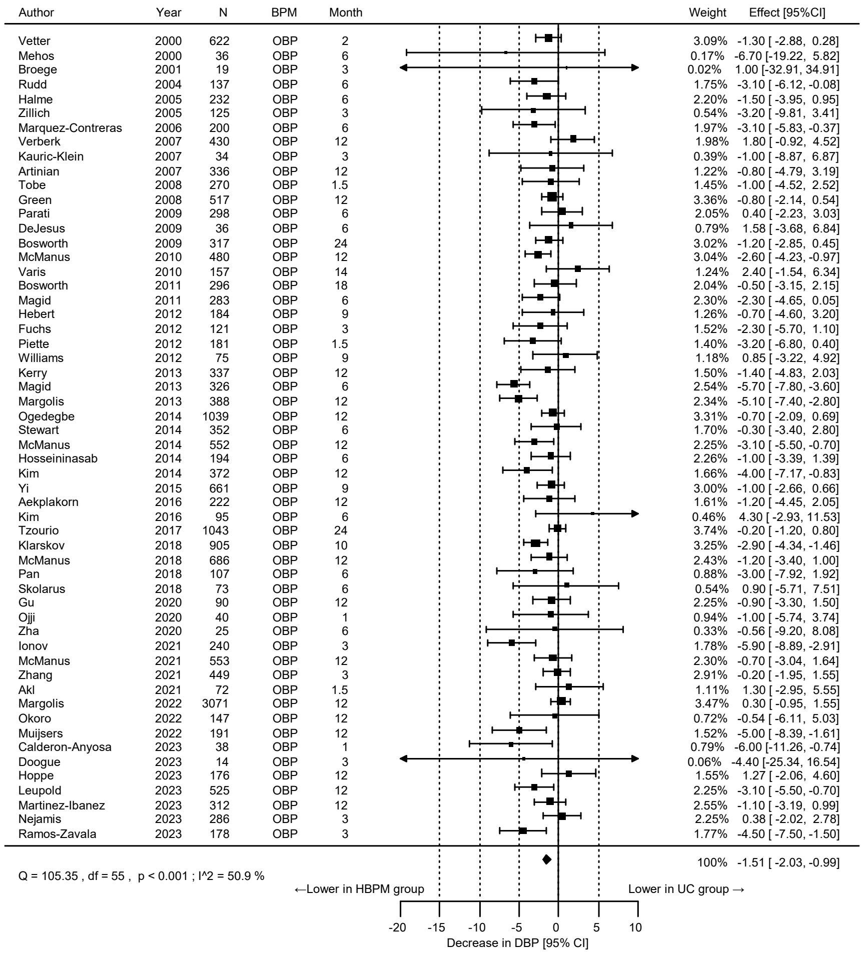

Outcome measure: Ambulatory BP (ABP)

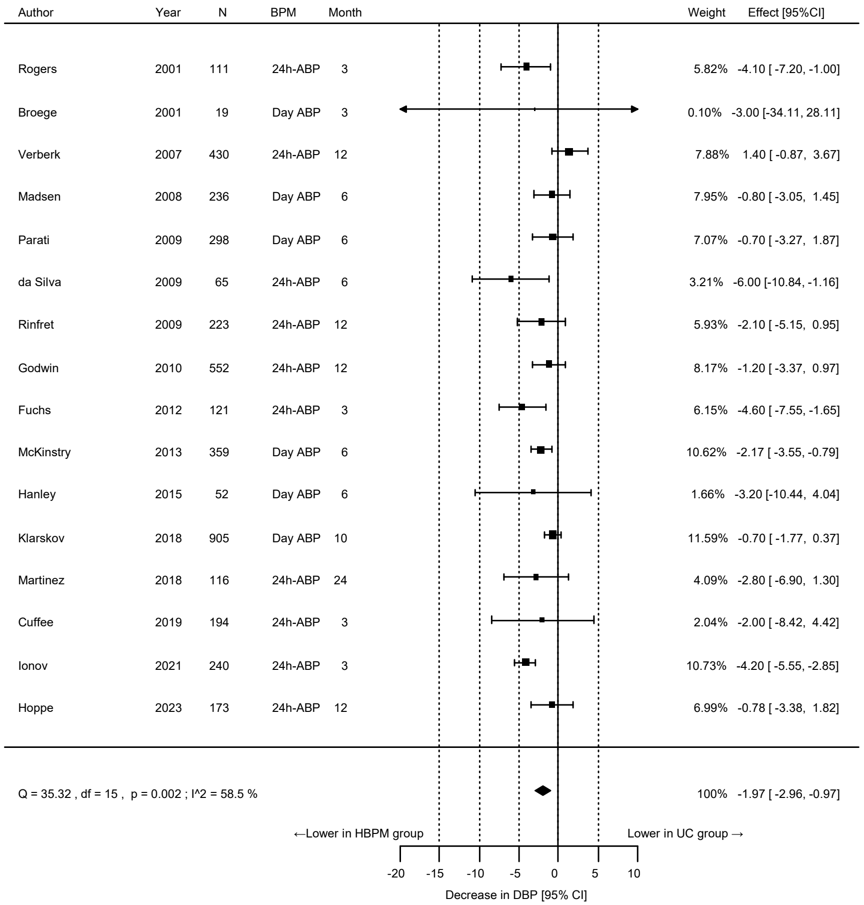

Supplementary Figure 5. The results for DBP change stratified by the type of outcome BP measurements

Abbreviations are the same as in **Supplementary Figure 4**.

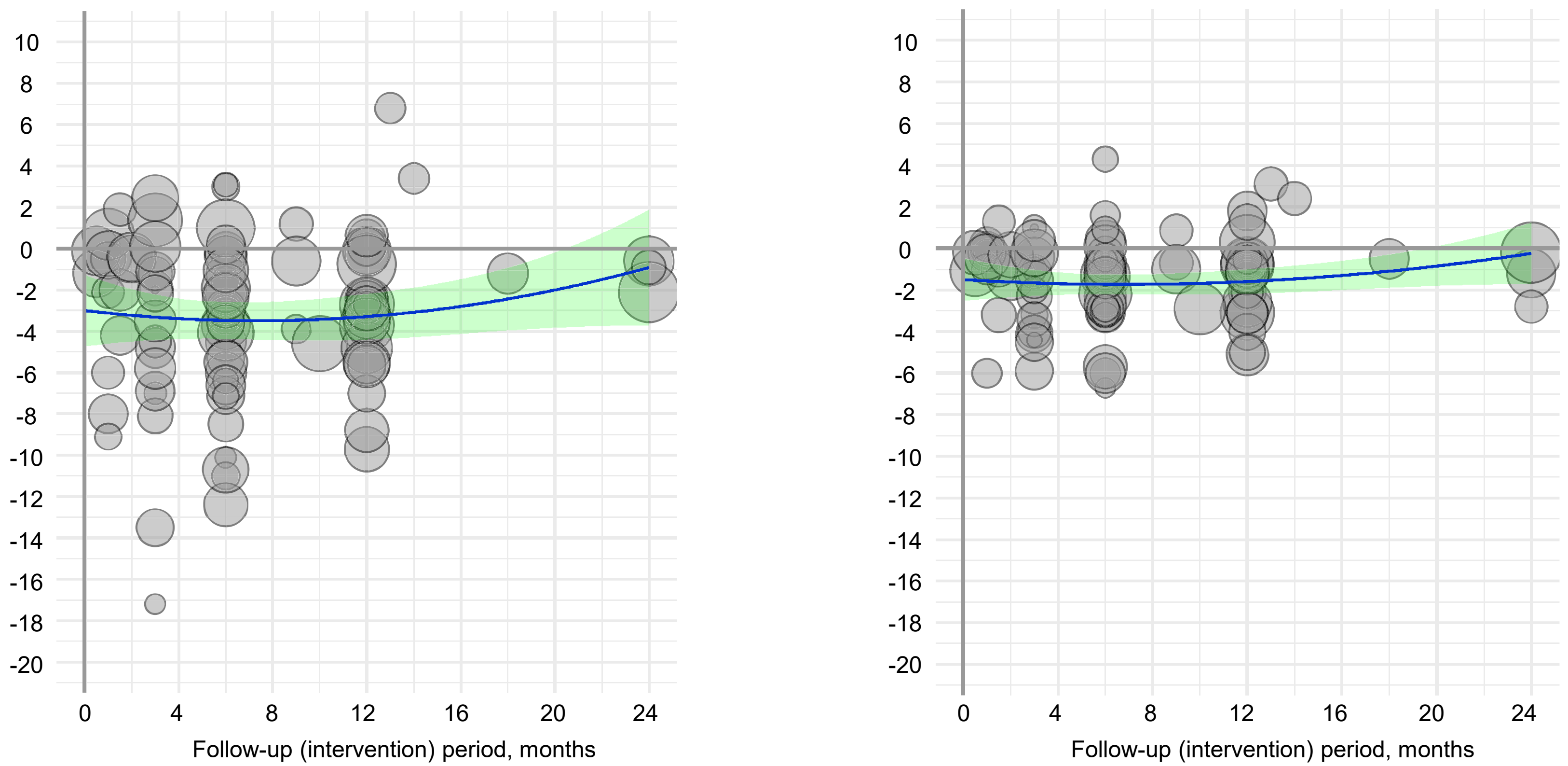

Supplementary Figure 6. The meta-regression analysis for the association between the follow-up period and the BP-lowering effect of HBPM

Likelihood ratio tests between a model that only included the follow-up period as a linear equation and a model that further included a quadratic equation showed that the latter was better (*p*=0.018 for SBP and *p*=0.038 for DBP). The size of each bubble represents the inverse of the standard error for the corresponding study.

Abbreviations are the same as in **Supplementary Figure 4**.

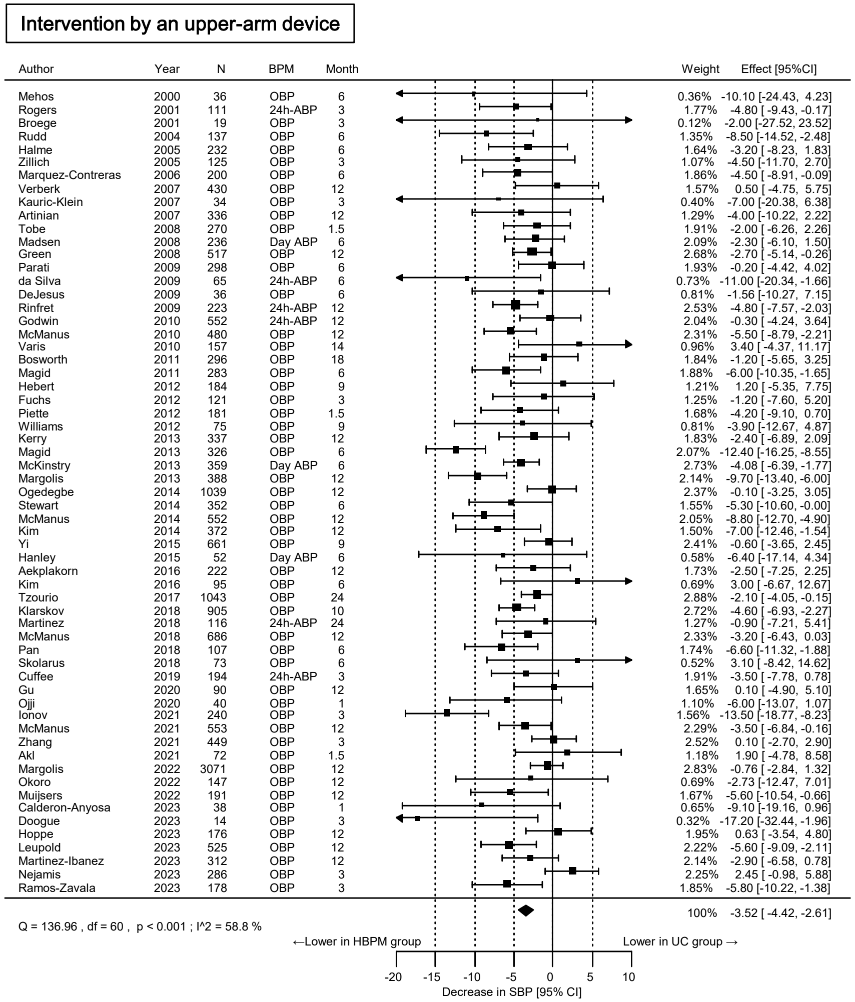

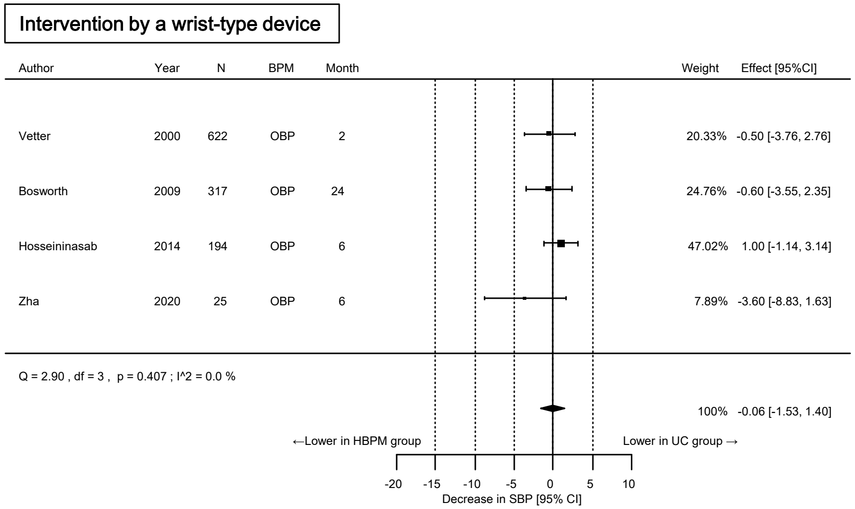

Supplementary Figure 7. The results for SBP change stratified by the device type for the intervention

Abbreviations are the same as in **Supplementary Figure 4**.

Intervention by an upper-arm device

Author	Year	N	BPM	Month	Weight	Effect [95%CI]
Mehos	2000	36	OBP	6	0.16%	-6.70 [-19.22, 5.82]
Rogers	2001	111	24h-ABP	3	1.61%	-4.10 [-7.20, -1.00]
Broege	2001	19	OBP	3	0.02%	1.00 [-32.91, 34.91]
Rudd	2004	137	OBP	6	1.66%	-3.10 [-6.12, -0.08]
Halme	2005	232	OBP	6	2.08%	-1.50 [-3.95, 0.95]
Zillich	2005	125	OBP	3	0.51%	-3.20 [-9.81, 3.41]
Marquez-Contreras	2006	200	OBP	6	1.86%	-3.10 [-5.83, -0.37]
Verberk	2007	430	OBP	12	1.87%	1.80 [-0.92, 4.52]
Kauric-Klein	2007	34	OBP	3	0.37%	-1.00 [-8.87, 6.87]
Artinian	2007	336	OBP	12	1.15%	-0.80 [-4.79, 3.19]
Tobe	2008	270	OBP	1.5	1.37%	-1.00 [-4.52, 2.52]
Madsen	2008	236	Day ABP	6	2.25%	-0.80 [-3.05, 1.45]
Green	2008	517	OBP	12	3.16%	-0.80 [-2.14, 0.54]
Parati	2009	298	OBP	6	1.94%	0.40 [-2.23, 3.03]
da Silva	2009	65	24h-ABP	6	0.86%	-6.00 [-10.84, -1.16]
DeJesus	2009	36	OBP	6	0.75%	1.58 [-3.68, 6.84]
Rinfret	2009	223	24h-ABP	12	1.64%	-2.10 [-5.15, 0.95]
Godwin	2010	552	24h-ABP	12	2.32%	-1.20 [-3.37, 0.97]
McManus	2010	480	OBP	12	2.87%	-2.60 [-4.23, -0.97]
Varis	2010	157	OBP	14	1.17%	2.40 [-1.54, 6.34]
Bosworth	2011	296	OBP	18	1.92%	-0.50 [-3.15, 2.15]
Magid	2011	283	OBP	6	2.17%	-2.30 [-4.65, 0.05]
Hebert	2012	184	OBP	9	1.19%	-0.70 [-4.60, 3.20]
Fuchs	2012	121	OBP	3	1.43%	-2.30 [-5.70, 1.10]
Piette	2012	181	OBP	1.5	1.33%	-3.20 [-6.80, 0.40]
Williams	2012	75	OBP	9	1.12%	0.85 [-3.22, 4.92]
Kerry	2013	337	OBP	12	1.41%	-1.40 [-4.83, 2.03]
Magid	2013	326	OBP	6	2.39%	-5.70 [-7.80, -3.60]
McKinstry	2013	359	Day ABP	6	3.12%	-2.17 [-3.55, -0.79]
Margolis	2013	388	OBP	12	2.21%	-5.10 [-7.40, -2.80]
Ogedegbe	2014	1039	OBP	12	3.11%	-0.70 [-2.09, 0.69]
Stewart	2014	352	OBP	6	1.61%	-0.30 [-3.40, 2.80]
McManus	2014	552	OBP	12	2.12%	-3.10 [-5.50, -0.70]
Kim	2014	372	OBP	12	1.56%	-4.00 [-7.17, -0.83]
Yi	2015	661	OBP	9	2.83%	-1.00 [-2.66, 0.66]
Hanley	2015	52	Day ABP	6	0.43%	-3.20 [-10.44, 4.04]
Aekplakorn	2016	222	OBP	12	1.52%	-1.20 [-4.45, 2.05]
Kim	2016	95	OBP	6	0.44%	4.30 [-2.93, 11.53]
Tzourio	2017	1043	OBP	24	3.52%	-0.20 [-1.20, 0.80]
Klarskov	2018	905	OBP	10	3.06%	-2.90 [-4.34, -1.46]
Martinez	2018	116	24h-ABP	24	1.10%	-2.80 [-6.90, 1.30]
McManus	2018	686	OBP	12	2.30%	-1.20 [-3.40, 1.00]
Pan	2018	107	OBP	6	0.84%	-3.00 [-7.92, 1.92]
Skolarus	2018	73	OBP	6	0.51%	0.90 [-5.71, 7.51]
Cuffee	2019	194	24h-ABP	3	0.54%	-2.00 [-8.42, 4.42]
Gu	2020	90	OBP	12	2.12%	-0.90 [-3.30, 1.50]
Ojji	2020	40	OBP	1	0.89%	-1.00 [-5.74, 3.74]
Ionov	2021	240	OBP	3	1.68%	-5.90 [-8.89, -2.91]
McManus	2021	553	OBP	12	2.17%	-0.70 [-3.04, 1.64]
Zhang	2021	449	OBP	3	2.74%	-0.20 [-1.95, 1.55]
Akl	2021	72	OBP	1.5	1.05%	1.30 [-2.95, 5.55]
Margolis	2022	3071	OBP	12	3.26%	0.30 [-0.95, 1.55]
Okoro	2022	147	OBP	12	0.68%	-0.54 [-6.11, 5.03]
Muijsers	2022	191	OBP	12	1.44%	-5.00 [-8.39, -1.61]
Calderon-Anyosa	2023	38	OBP	1	0.75%	-6.00 [-11.26, -0.74]
Doogue	2023	14	OBP	3	0.06%	-4.40 [-25.34, 16.54]
Hoppe	2023	176	OBP	12	1.47%	1.27 [-2.06, 4.60]
Leupold	2023	525	OBP	12	2.12%	-3.10 [-5.50, -0.70]
Martinez-Ibanez	2023	312	OBP	12	2.40%	-1.10 [-3.19, 0.99]
Nejamis	2023	286	OBP	3	2.12%	0.38 [-2.02, 2.78]
Ramos-Zavala	2023	178	OBP	3	1.67%	-4.50 [-7.50, -1.50]
					100%	-1.65 [-2.16, -1.15]

Q = 113.89 , df = 60 , p < 0.001 ; I^2 = 49.9 %

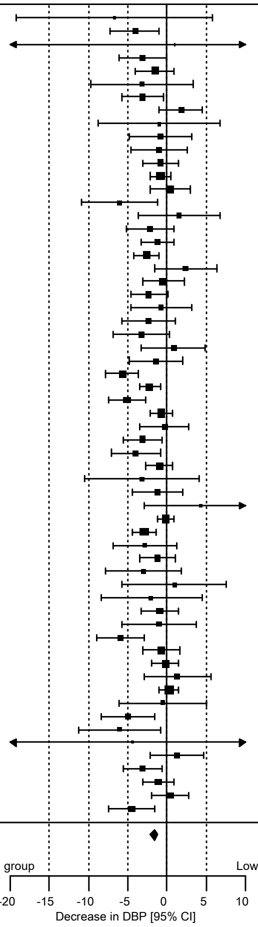

Intervention by a wrist-type device

Author	Year	N	BPM	Month	Weight	Effect [95%CI]
Vetter	2000	622	OBP	2	41.73%	-1.30 [-2.88, 0.28]
Bosworth	2009	317	OBP	24	38.50%	-1.20 [-2.85, 0.45]
Hosseininasab	2014	194	OBP	6	18.37%	-1.00 [-3.39, 1.39]
Zha	2020	25	OBP	6	1.40%	-0.56 [-9.20, 8.08]
					100%	-1.20 [-2.22, -0.17]

Q = 0.06 , df = 3 , p = 0.996 ; I^2 = 0.0 %

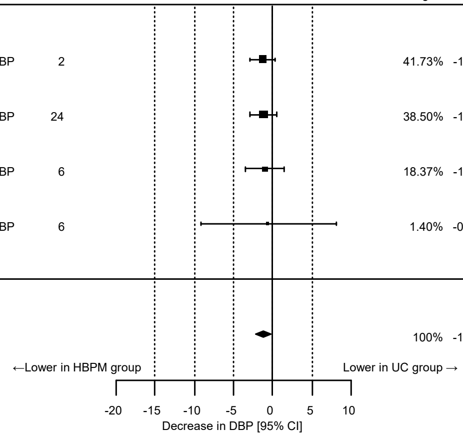

Supplementary Figure 8. The results for DBP change stratified by the device type for the intervention

Abbreviations are the same as in **Supplementary Figure 4**.

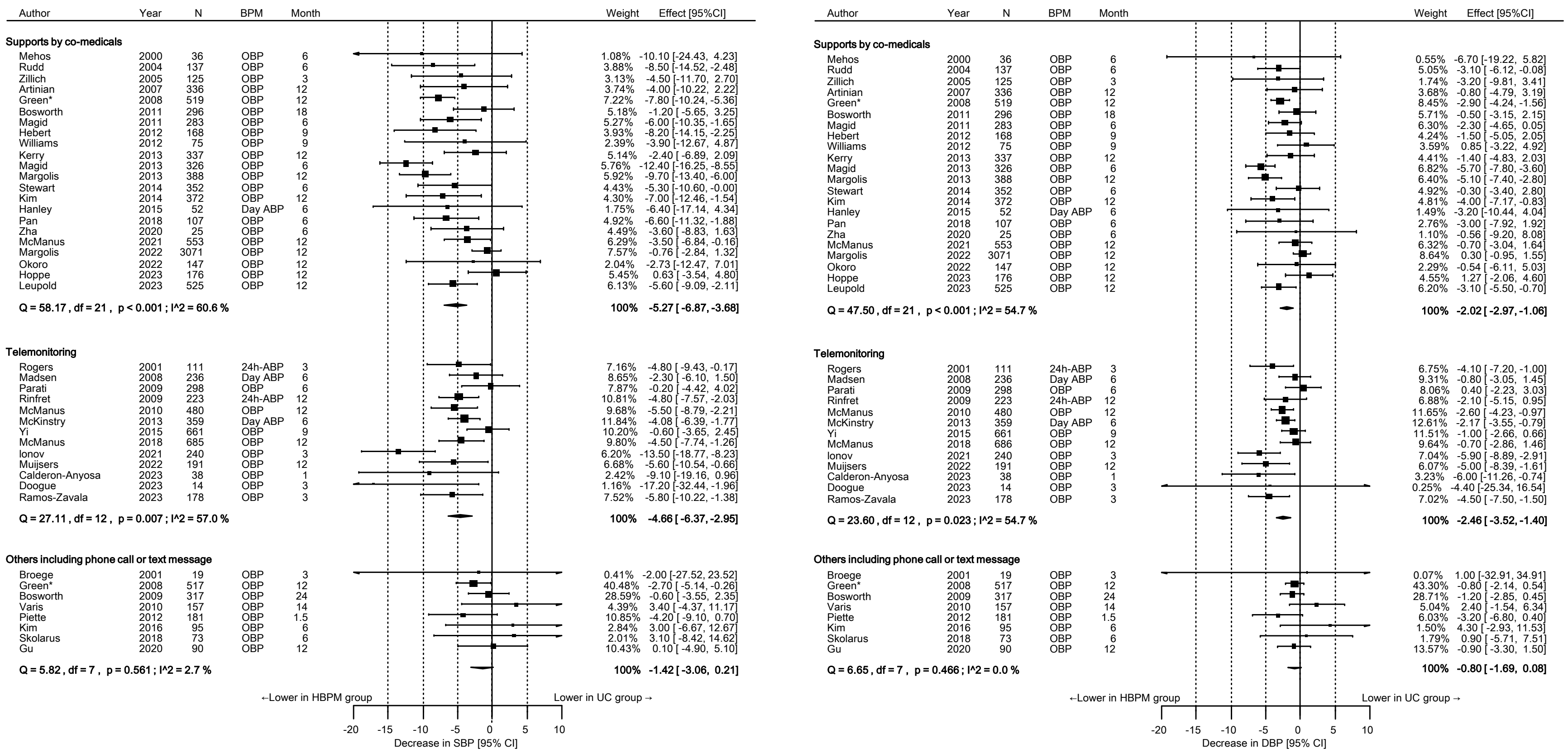

Supplementary Figure 9. The results stratified by the type of co-intervention

Abbreviations are the same as in **Supplementary Figure 4**.

*The trial by Green et al. had three arms.

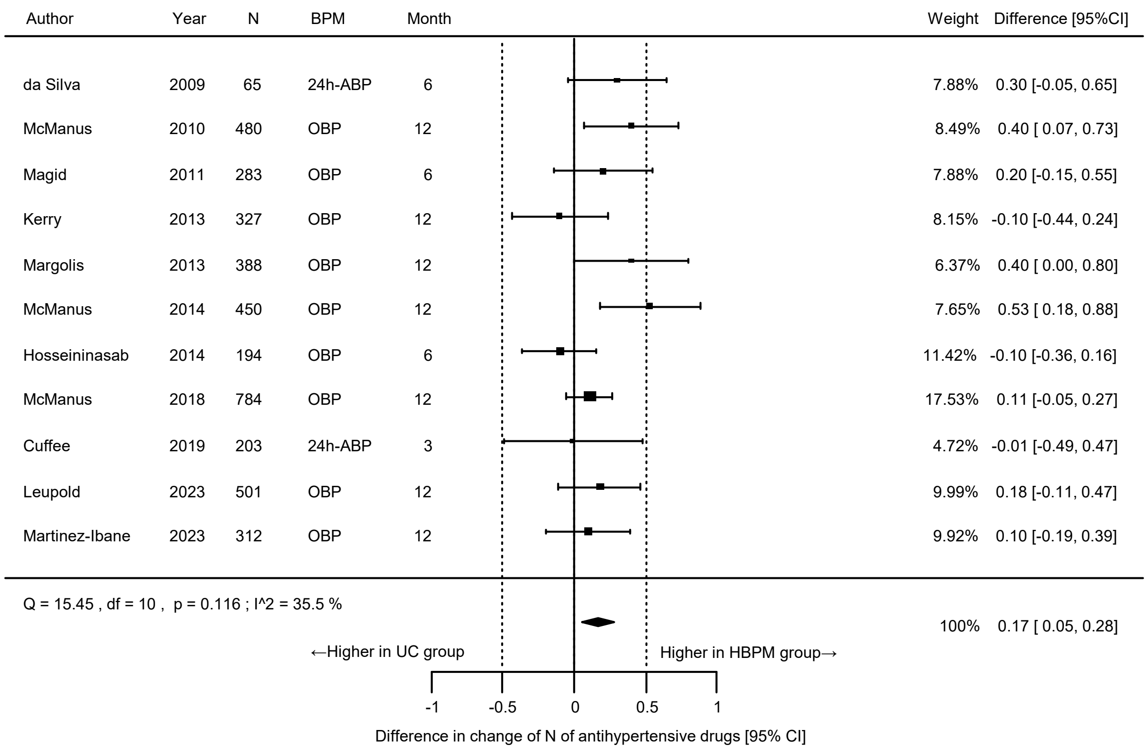

Supplementary Figure 10. Difference in antihypertensive drug change at the end of intervention between groups

Abbreviations are the same as in **Supplementary Figure 4**.

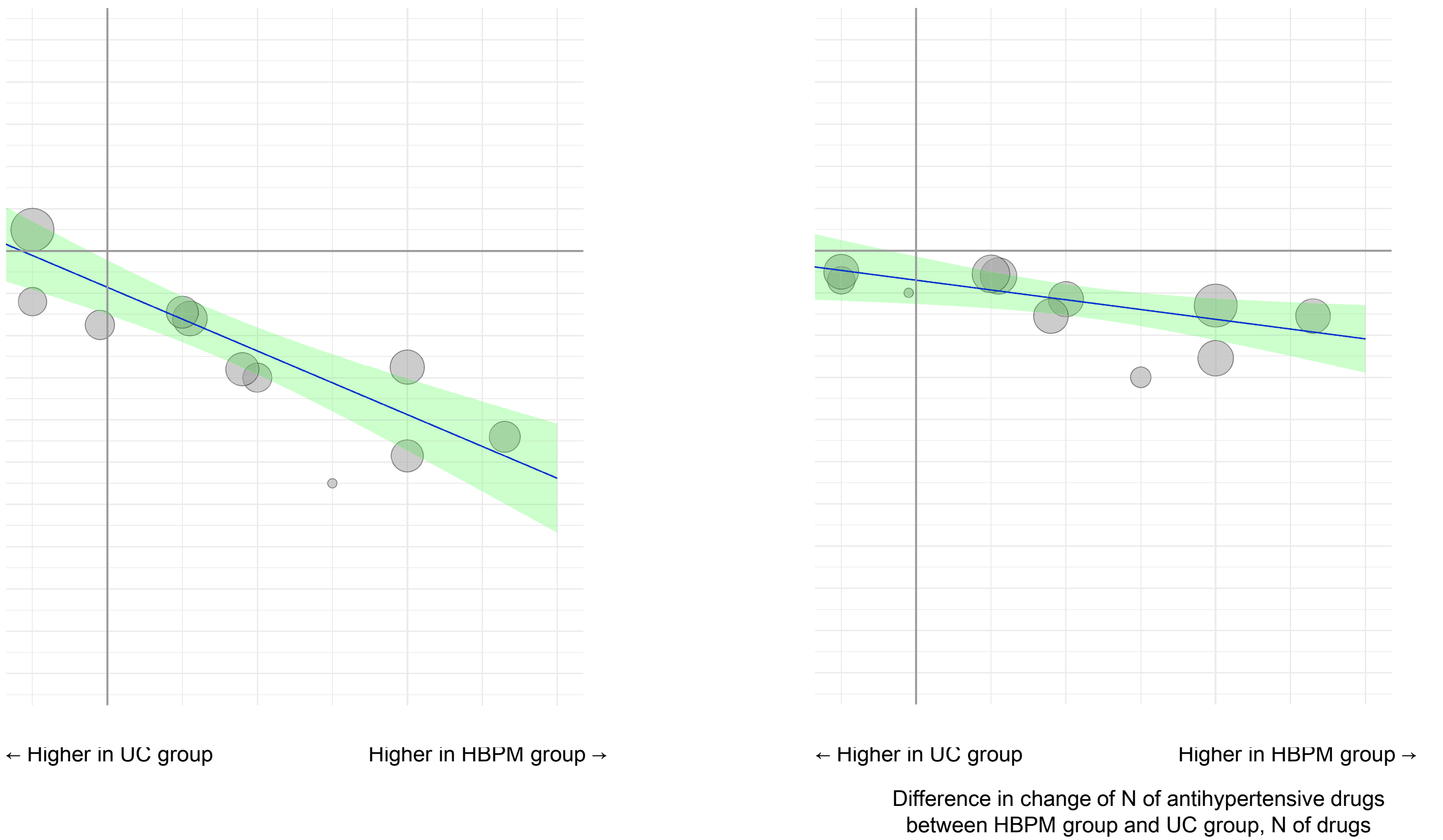

Supplementary Figure 11. The meta-regression analysis for the association between the difference in the change of the number of antihypertensive medications and the BP-lowering effect of HBPM

Abbreviations are the same as in **Supplementary Figure 4**. The size of each bubble represents the inverse of the standard error for the corresponding study.

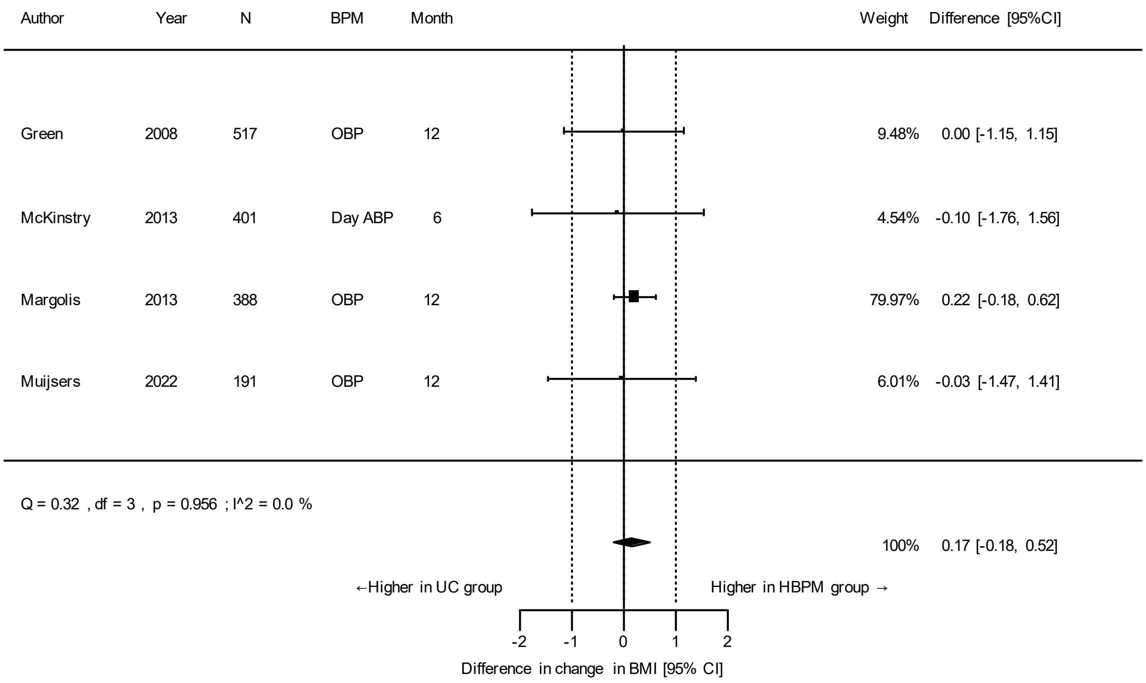

Supplementary Figure 12. The difference in BMI change between groups

BMI, body mass index. Other abbreviations are the same as those in **Supplementary Figure 4**.

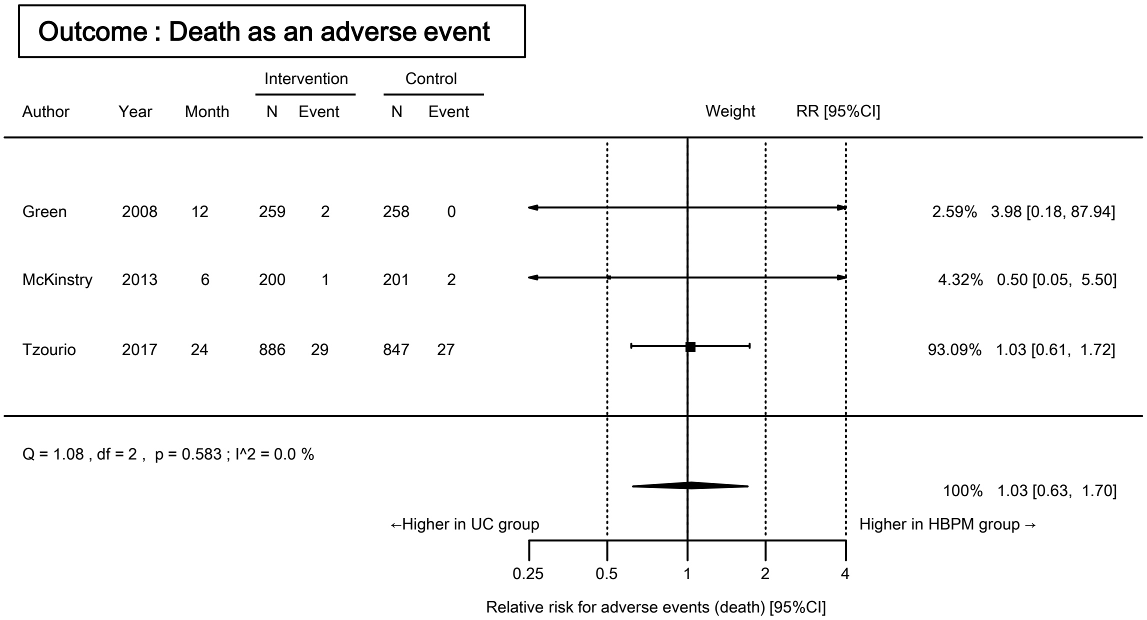

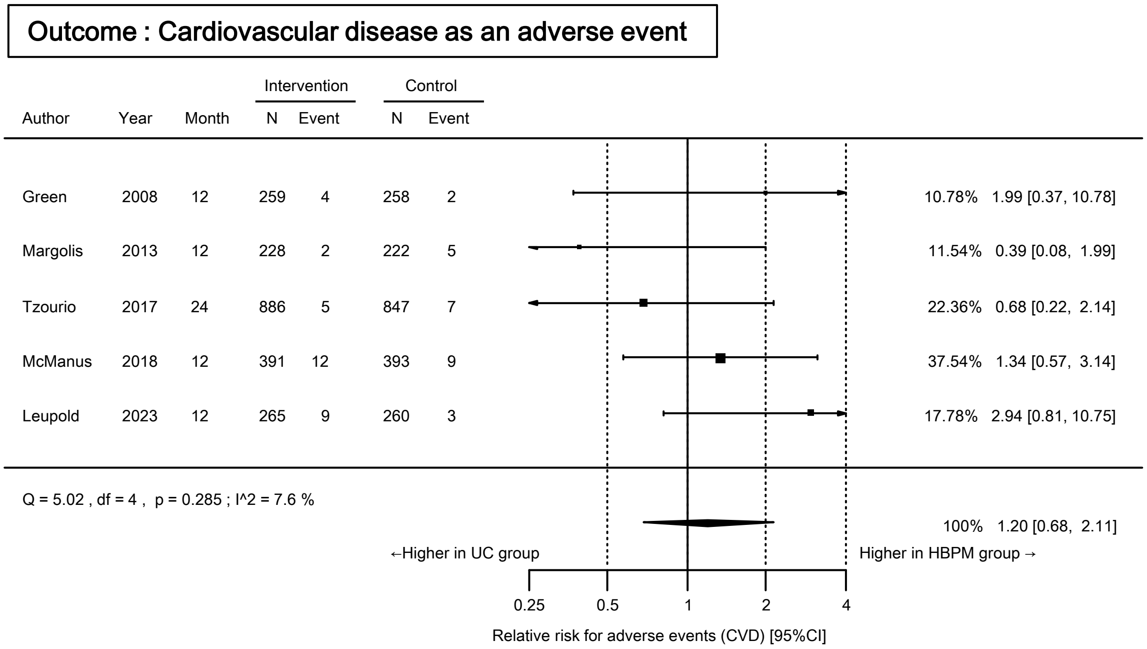

Supplementary Figure 13. Incidence proportion ratios of HBPM vs UC as a reference for severe adverse events

CVD, cardiovascular disease; RR, relative risk. Other abbreviations are the same as those in **Supplementary Figure 4**.
